# Clonality and non-linearity drive facultative-cooperation allele diversity

**DOI:** 10.1038/s41396-018-0310-y

**Published:** 2018-11-21

**Authors:** Ishay Ben-Zion, Shaul Pollak, Avigdor Eldar

**Affiliations:** 1 Faculty of Life Sciences, School of Molecular Cell Biology and Biotechnology, Tel-Aviv University, PO Box 39040, 6997801 Tel-Aviv, Israel; 2 Present Address: Department of Systems Biology, Harvard Medical School, Boston, MA 02115 USA; 3 Present Address: Department of Civil and Environmental Engineering, Massachusetts Institute of Technology, Cambridge, MA 02139-4307 USA

**Keywords:** Population genetics, Microbial ecology

## Abstract

Kin discrimination describes the differential interaction of organisms with kin versus non-kin. In microorganisms, many genetic loci act as effective kin-discrimination systems, such as kin-directed help and non-kin-directed harm. Another important example is facultative cooperation, where cooperators increase their investment in group-directed cooperation with the abundance of their kin in the group. Many of these kin-discrimination loci are highly diversified, yet it remains unclear what evolutionary mechanisms maintain this diversity, and how it is affected by population structure. Here, we demonstrate the unique dependence of kin-discriminative interactions on population structure, and how this could explain facultative-cooperation allele-diversity. We show mathematically that low relatedness between microbes in non-clonal social groups is needed to maintain the diversity of facultative-cooperation alleles, while high clonality is needed to stabilize this diversity against cheating. Interestingly, we demonstrate with simulations that such population structure occurs naturally in expanding microbial colonies. Finally, analysis of experimental data of quorum-sensing mediated facultative cooperation, in *Bacillus subtilis*, demonstrates the relevance of our results to realistic microbial interactions, due to their intrinsic non-linear frequency dependence. Our analysis therefore stresses the impact of clonality on the interplay between exploitation and kin discrimination and portrays a way for the evolution of facultative cooperation.

## Introduction

Microorganisms engage in numerous social behaviors that affect their neighbors, ranging from helping to harming behaviors. The ability to interact differentially with genetic relatives versus non-relatives, called kin discrimination, is therefore important. In microorganisms, kin-discrimination systems are abundant and often display high allelic diversity [1]. Examples include non-kin harming systems (e.g., bacteriocin-immunity pairs [2, 3], type VI secretion [4], or contact-dependent inhibition [5]), and kin-directed helping systems (e.g., siderophore-receptor pairs [6, 7], and contact-dependent cooperative behaviors [8–11]).

A third, ubiquitous, example is facultative cooperation (also designated facultative cheating [12–17] or strategic cooperation [18]). In this situation, organisms increase their level of cooperation with the abundance of their kin in their interaction neighborhood, without specifically directing cooperation to their kin. This occurs in amoeba and bacteria, which were shown to reduce their cooperative efforts during fruiting body formation in mixed-genotype groups compared to clonal groups [12, 18]. In addition, the density-dependent control of many bacterial cooperative behaviors by cell-cell signaling, also known as quorum sensing (QS), often leads to facultative cooperation. This is because the cooperative effort of a strain positively depends on the concentration of QS signaling molecules produced by kin with a similar QS allele [19, 20]. We recently showed that mutual facultative cooperation occurs between strains coding for divergent QS receptor-signal alleles (also known as pherotypes). In these pherotypes, each QS receptor responds specifically to its co-encoded signal, leading to a situation where the minority pherotype senses less of its signal and thus cooperates less than the majority pherotype while the benefit is shared by both [16, 17, 21]. Facultative-cooperation divergent alleles therefore follow a negative frequency-dependent selection (i.e., minority wins), in contrast to other, directed, kin-discrimination alleles that show a positive frequency-dependent selection (i.e., majority wins) [1].

It is generally unknown what mechanisms maintain allele diversity in microbial kin-discrimination systems [1]. For facultative-cooperation alleles, negative frequency dependence ensures coexistence of divergent alleles in unstructured populations, but in such conditions, facultative cooperators face invasion by cheating alleles that will eliminate diversity [16, 21]. Furthermore, microbial populations in the wild are often structured such that individuals do not interact randomly with other individuals, but mostly interact with their kin in locally-clonal groups. This happens either because of extreme bottlenecks occurring when entering an unoccupied niche [22–24], or because of the aggregatory mode of growth of microbes with limited motility in colonies or biofilms [25–28]. A simple metric for population structure, originally defined by Hamilton [29], is the genetic relatedness between interacting organisms, which captures the probability of an organism to interact with organisms of the same genetic makeup in a relevant locus.

In this work, we wish to understand the role of population structure in selection on divergent kin-discrimination alleles in general, and specifically on divergent facultative-cooperation variants. The effect of population structure on the maintenance of cooperation in the face of cheating is theoretically rather clear [22, 29–32]; cheaters outcompete cooperators within mixed groups, whereas competition between groups, and prominently between clonal groups, works in the favor of cooperators. The interplay between these two forces is governed by population structure, as was famously summarized in Hamilton’s rule [29]; *rb* > *c*, where *b,c* are measures of the benefit and cost of a cooperative interaction and *r* is the relatedness measure [33]. In contrast to cooperation, the effect of population structure on interactions between kin-discrimination variants, which we term here kin-discriminative interactions, is less clear. In such interactions, clonal groups of organisms carrying different kin-discrimination alleles are expected to behave similarly and have the same fitness, resulting in an inter-group competition effect that is more complex [34].

Here, we address this problem, focusing on the impact of the strong clonality of microbial populations on kin-discriminative interactions and specifically on facultative cooperation. To this aim, we combine three approaches. First, we formalize a Hamilton-like rule that separately analyzes the clonal and non-clonal social neighborhoods in a structured population. Consequently, we show that facultative-cooperation allele diversity can be maintained concomitantly with elimination of cheaters in a population structure with high level of clonality and low relatedness in non-clonal groups. Second, using simulations, we show how this theoretical understanding applies to two realistic, highly clonal, microbial population structures derived from expansion through extreme bottlenecks and colony growth. Finally, we analyze experimental data of competitions between QS variants in *Bacillus subtilis* to show the relevance of our predictions to realistic microbial interactions due to their intrinsic non-linearity.

## Results

### A Hamilton-like invasion rule for kin-discriminative interactions suggests conditions for the maintenance of facultative-cooperation allele diversity

To consider the role played by population structure in the evolution of kin-discrimination allelic diversity, we first utilized the general analytical approach of the Price equation [35] to rewrite Hamilton’s rule in a form that decouples clonal from non-clonal effects on fitness, *W* (see Methods and SI text for restrictions and derivations). Specifically, we find that the condition for invasion of a rare genotype (denoted by index 1) into a resident population of a common genotype (denoted by index 2) can be written as a weighted sum of two terms:

1 $$x_{1c} \cdot \underbrace {\left( {\left\langle W \right\rangle _{1c} - \left\langle W \right\rangle _{2c}} \right)}_{{\mathrm{\Delta }}W_{\mathrm{{clonal}}}} + \left( {1 - x_{1c}} \right) \cdot \underbrace {\left( {\rho b_{\mathrm{{nc}}} - c_{\mathrm{{nc}}}} \right)}_{{\mathrm{\Delta }}W_{\mathrm{{non - clonal}}}} > \, 0.$$

**Fig. 1:**
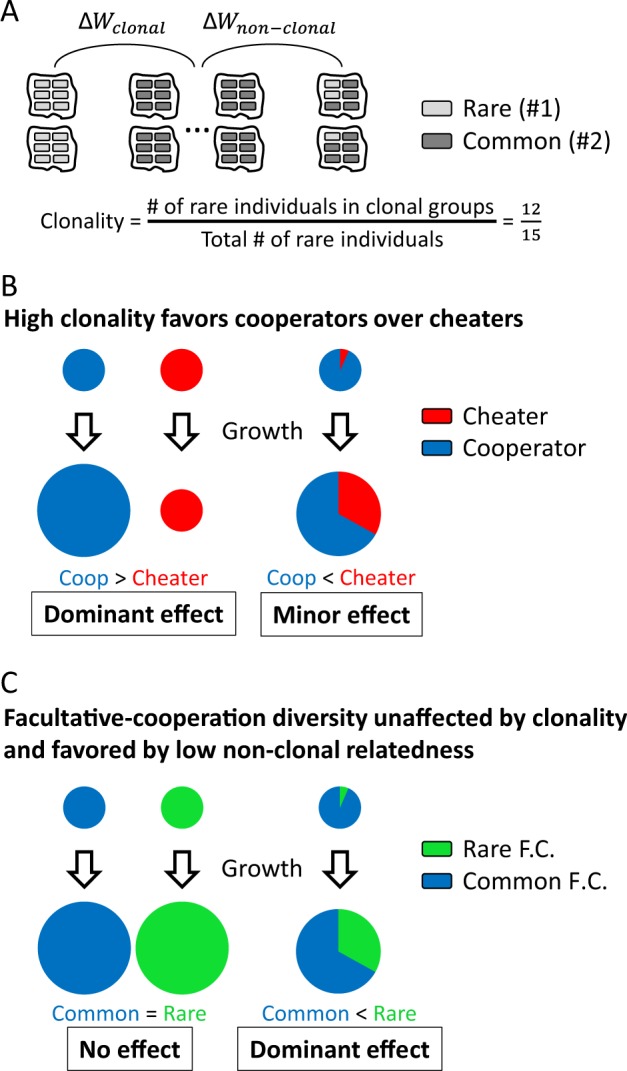
Highly clonal structured population selects for facultative-cooperation allele diversity and against cheaters. **a** A schematic description of a structured population composed of purely clonal socially interacting groups and mixed-genotype groups. The fate of a rare genotype would be dictated by how well it does in clonal and non-clonal groups compared to clonal groups of the common genotype (Eq. 1). Clonality level, *x*_*1*__*c*_, is defined as the fraction of the rare genotype individuals that reside in strictly clonal groups. **b**, **c** Pie chart representation of the fate of different social interactions in structured populations with high clonality. Top and bottom pie charts represent start and final populations, with the pie size representing total population size and color slices representing frequencies. **b** In exploitive interactions, a clonal cheater group (red) has lower fitness (growth factor) than a clonal cooperator group (blue), while cheaters have higher fitness than cooperators in mixed groups. If clonality is high, selection will be dominated by clonal social groups. **c** Two kin-discrimination alleles, e.g., facultative-cooperation (F.C.) variants, have equal fitness in clonal groups, but different fitness in mixed groups. Direction of selection will be affected only by the non-clonal groups, irrespective of clonality level. Facultative-cooperation alleles display negative-frequency dependent selection in well-mixed conditions. Therefore, if the rare variant is in minority in its social interaction groups (i.e., if non-clonal relatedness is low), then it will increase in frequency in the total (structured) population

These two terms represent selection on the clonal and non-clonal subpopulations of the rare genotype compared to the baseline fitness of clonal groups of the common genotype (Fig. 1a). The weighting factor, *x*_1*c*_, which we denote as clonality level, is the fraction of genotype #1 individuals that are in clonal social neighborhoods (Fig. 1a). The first term is the difference between the mean fitness of the two genotypes in their clonal groups (〈*W*〉_1*c*_ and 〈*W*〉_2*c*_). The second term is written in a Hamilton rule-like form, where the measures *ρ*, *b*_nc_, *c*_nc_ are defined as *r,b,c* in Hamilton’s rule, respectively, but taking into account only genotype #1 individuals that reside in non-clonal groups (Fig. 1a and SI text). We therefore term *ρ* as “non-clonal relatedness”. Importantly, its value can be very different than that of the general relatedness, *r* (see Box 1 and SI text).

The partition of the invasion condition in Eq. 1 is particularly useful for contrasting the effect of clonality on exploitive interactions (i.e., between cooperators and cheaters) (Fig. 1b), versus its effect on kin-discriminative interactions, e.g., between facultative-cooperation variants (Fig. 1c). High level of clonality strongly favors cooperation over cheating since clonal groups of cooperators have a higher fitness than clonal groups of cheaters and since the effect of the intra-group advantage of cheaters is minor in this case (Fig. 1b). In contrast, clonal groups of different kin-discrimination variants have the same fitness (Fig. 1c): ΔW_clonal _= 0, and Eq. 1 is reduced to a Hamilton rule for genotype #1 individuals in non-clonal groups:

2 $$\rho b_{\mathrm{{nc}}} - c_{\mathrm{{nc}}}\hskip 2pt > \hskip 2pt 0.$$

Kin-discriminative interactions therefore do not depend on the clonality level, but depend only on the social effects occurring on the non-clonal sub-population of the rare genotype (Fig. 1c and Box 1).

How would this understanding apply to interactions between different facultative-cooperation variants and the corresponding cheater variants? As explained above, cheaters would be eliminated if clonality is sufficiently high. Since different facultative-cooperation variants display negative frequency-dependent selection, the frequency of a minority allele (in the total population) will increase if it is also a minority in mixed-genotype social groups, i.e., if non-clonal relatedness is low (Fig. 1c, see Box 1 and SI text). To summarize, a population structure with high clonality and low non-clonal relatedness will promote the coexistence of facultative-cooperation alleles, while avoiding invasion of cheaters.

Box 1 Hamilton’s rule for kin-discriminative interactions – a mathematical expansion Eq. 2 has a similar structure as that of Hamilton’s rule, but it takes into account only the non-clonal sub-population of the invading genotype. This brings forward the question of what will be the relation between population structure and frequency-dependent fitness in this form compared to the general Hamilton rule.

**Relatedness measures**: In the limit where genotype #1 is rare, the general relatedness coefficient *r* is given by the mean probability for a genotype #1 individual to find genotype #1 individuals (including itself) in its social environment (see ref. [47] and SI text). For example, in Fig. 1a it is $r =\frac{1}{15}\cdot ({{12 \cdot 1 + 2 \cdot \frac{2}{6} + 1 \cdot \frac{1}{6}}}) = 0.86$. Non-clonal relatedness, *ρ*, is similarly defined but only for invading individuals in non-clonal groups. Thus in Fig. 1a it is $\rho = \frac{1}{3} \cdot ({{2 \cdot \frac{2}{6} + 1 \cdot \frac{1}{6}}}) = 0.28\hskip 2pt < \hskip 2pt r$. The relation between general relatedness, non-clonal relatedness and clonality (which in Fig. 1a is $x_{1c} = \frac{{12}}{{15}}$) is given by (see SI text):

3 $$r = x_{1c} + \left( {1 - x_{1c}} \right)\rho .$$

Therefore, a high level of general relatedness *r* can occur if the level of clonality *x*_1*c*_ is high, even if non-clonal relatedness *ρ* is low.

**Effects of non-linearity on selection:** In addition to its dependence on the non-clonal relatedness *ρ*, Hamilton’s rule for kin-discriminative interactions is affected by the frequency dependence of the invader’s fitness function, *W*_1_, which, in microorganisms, could be highly non-linear [47]. In the SI text, we show that under the simplifying assumptions that the invader fitness is continuous and depends only on its frequency, two qualitatively different selection patterns can arise. If the global minimum (maximum) of the invader fitness occurs at clonal groups then invasion will always succeed as in Box Fig. A (always fail as in Box Fig. C), independently of population structure. Specifically, this means that if an interaction between kin-discrimination genotypes is linear, then its fate would not depend on population structure. In contrast, if a global extremum of the fitness occurs at an intermediate frequency, then population structure, and specifically non-clonal relatedness *ρ*, could affect the direction of selection (Box Fig. B, D). For the case of negative frequency-dependent selection between facultative-cooperation variants, we show in the SI text that under realistic assumptions the fitness of each variant will have a minimum at an intermediate frequency. This implies that invasion of each allele will occur if the non-clonal relatedness *ρ* is low enough (Box Fig. B, SI text). For the case of positive-frequency dependent selection, invasion will succeed when non-clonal relatedness is high (Box Fig. D). The above statements are easily understood by considering the invasion condition as a comparison between the invader's mean fitness (over all social neighborhoods) and the resident's clonal fitness (SI text).

**The shape of the frequency-dependent selection function determines the impact of population structure**. Examples for fitness functions for kin-discriminative interactions between an invader (green) and a resident (blue), made symmetrical for simplicity. Dashed lines demonstrate equal clonal fitness. Shown are cases for negative (**a**, **b**) and positive (**c, d**) frequency-dependent selection. (**a**, **c**) If the clonal invader fitness is the global minimum (**a**) (maximum (**c**)) of the invader’s fitness function, then invasion will always succeed (**a**) (fail (**c**)), irrespective of the population structure. (**b, d**) If fitness of clonal groups is not a global extremum, then population structure would affect invasion, and invasion would succeed at low (**b**) (high (**d**)) non-clonal relatedness *ρ*. We note that success of invasion of a rare genotype does not depend on the residents full fitness function, but only on its clonal fitness.

### Facultative-cooperation divergent alleles coexist and resist cheating in realistic microbial structured populations

In the above section, we theoretically showed that maintenance of facultative-cooperation alleles and their resistance to invasion by cheaters could be accomplished in a structured population with low non-clonal relatedness and high level of clonality. As with other applications of Hamilton’s rule, applying this insight to realistic scenarios requires an understanding of the way by which growth, competition and population structure are coupled with fitness [33]. We therefore explored the behavior of microbial facultative-cooperation strategies in two common models of population structure, which emulate the two processes by which microbes obtain high clonality – strong bottlenecks and expansion during colonial growth. In both cases, we assumed that social interactions weakly affect microbial growth (i.e., fecundity), but that population dynamics and fitness may also be affected by other components of the life cycle, such as local competition, migration or drift.

**Fig. 2:**
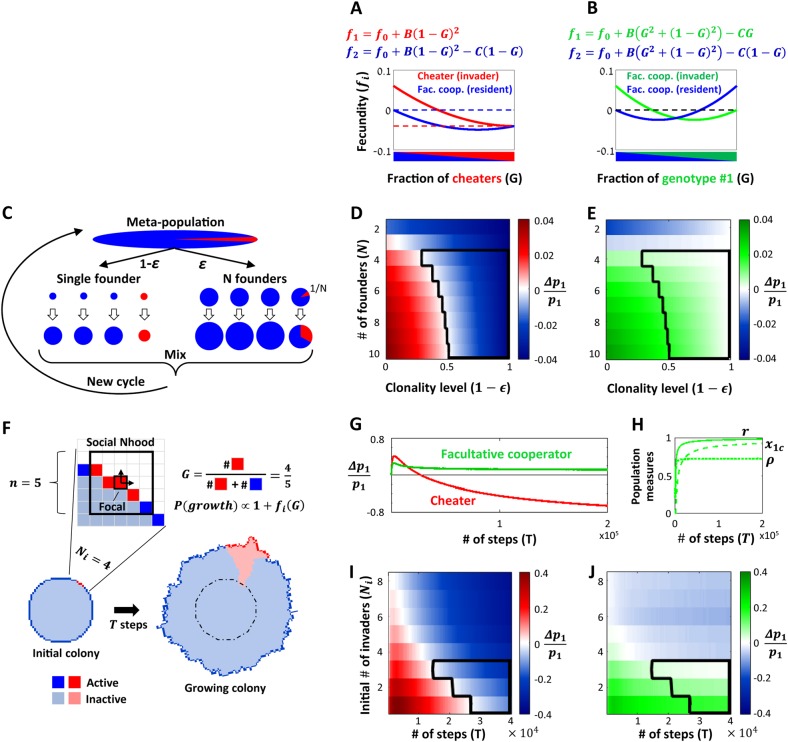
Comparing exploitive and kin-discriminative interactions in two population structures with high clonality. **a**, **b** The fecundity of two interacting strains in a well-mixed interaction group with a fraction *G* of invaders. Shown are the equations and graphs for the fecundity functions for the interaction between a facultative cooperator strain and a cheater strain (**a**) and between two facultative-cooperation strains (**b**). The basal fecundity parameter *f*_0_ is chosen such that *f*_2_(*G* = 0) = 0. Dashed lines emphasize clonal fitness values. **c**–**e** Infinite island model with both clonal and non-clonal bottlenecks, with full migration. **c** The simulation scheme, as described in the text (pie chart representation as in Fig. 1b). **d**, **e** Relative change in the invader’s frequency (*p*_1_) for varying values of the large bottleneck size (*N*) and the clonality level (1−*∈*), for the two interactions in panels **a**, **b**, respectively. The outlined area denotes conditions that permit mutual invasion of facultative cooperators but prevent cheater invasion. The parameters for these two plots are *B* = 0.1 and *C* = 0.06. **f**–**j** Social selection in colony growth simulations. **f** A scheme of a colony growth simulation. Growth probability of a focal active microbe on the edge of the colony depends on the relative frequency *G* of active microbes in a defined neighborhood around the focal microbe. In the example, neighborhoods size is: *n* = 5, and initial invader cluster size is: *N*_*i*_ = 4. **g** The relative change in invader frequency for a cheater (red), or facultative cooperator (green), invading a resident facultative cooperator. The initial number of invaders is *N*_*i*_ = 1. **h** Population structure measures (general relatedness *r* (solid line), non-clonal relatedness *ρ* (dotted line) and clonality level *x*_1*c*_ (dashed line)) for the mutual facultative cooperators data plotted in panel **g** (see SI text). **i**, **j** The relative change in invader frequency, for varying levels of the initial number of invaders (*N*_*i*_), and the number of time steps (*T*), for the two interactions in panels (**a**, **b**), respectively. The outlined area denotes conditions that permit mutual invasion of facultative cooperators but prevent cheater invasion. To maintain constant initial frequency in the meta-population of all repeats, a simulation with a given *N*_*i*_ had $\frac{{84,000}}{{N_i}}$ runs that started with *N*_*i*_ invading microbes on the colony border, while the rest of the runs started with zero invading microbes. The simulations in panels **g**–**j** was done using Matlab 2017a, with parameters: *n* = 11, *B* = 0.2, *C* = 0.12

To this aim, we consider the impact of the models on the exploitive interaction between a facultative cooperator and a cheater (Fig. 2a) and on the kin-discriminative interaction between strains carrying two variants of the facultative-cooperation allele (Fig. 2b). We assume that within an interacting group, facultative cooperators secrete a public good, in a manner proportional to their frequency in the population, and suffer a proportional fitness cost (*C*). The associated benefit (*B*) is proportional to the overall quantity of public goods produced. A cheater strain enjoys the public goods produced by cooperators without investing in their production.

#### Infinite island model with both clonal and non-clonal bottlenecks

We first consider the strong bottleneck scenario using an infinite island model with migration (e.g., [34, 36–38], see a more detailed description of the model in the SI text). Briefly, we study the change in frequency of two different strains; a common (resident) strain and a rare (invader) strain. Microbes of the two genotypes can inoculate an infinite number of growth patches of similar ecological context. To understand the impact of strict clonality on the process, we assume that a fraction ${\it{\epsilon }}$ of the microbes inoculates patches that allow inoculation by *N* founders and may therefore result in mixed-genotype patches, while the rest (1−${\it{\epsilon }}$) experience a stronger bottleneck and singly inoculate a smaller patch (of *N* = 1), which will therefore be strictly clonal. Each microbe in a patch first grows by a constant factor in an asocial manner until density is sufficiently high to introduce social effects on growth. Growth (fecundity) of each microbe, under social conditions, is dependent on its genotype and on the frequency of the two genotypes in the patch (Fig. 2a, b). To simulate possible mixing of patches we assume that after social growth, microbes can migrate to a new patch at a probability *m*, or remain in their patch, at a probability 1−*m*. Finally, a new cycle of growth arises by random sampling of each patch population back to its original inoculum size (either 1 or *N*). Figure 2c describes this model for the case of full migration (*m* = 1).

Based on our theoretical predictions, we expect selection for the cheater to depend on the general relatedness which will depend on the three parameters ${\it{\epsilon }}$,*N* and *m*. In contrast, the direction of selection for a kin-discriminative strategy should depend only on the interaction in non-clonal patches and therefore should depend on *N* and *m*, but not on ${\it{\epsilon }}$. A full analysis of this model (SI text) agrees well with these expectations. This is specifically simple for the case of full migration, where we find a simple formula for the general and non-clonal relatedness coefficients: $r = 1 - {\it{\epsilon }} + {\it{\epsilon }}\frac{1}{N},\,\rho = \frac{1}{N}$ and for clonality level *x*_1*c* _= 1−${\it{\epsilon }}$. These imply that cheater invasion succeeds when clonality level (1−${\it{\epsilon }}$) is small and the non-clonal inoculum size (*N*) is large (i.e., general relatedness is low) (Fig. 2d). In contrast, invasion of one facultative cooperator into a population of another succeeds when *N* is large (i.e., non-clonal relatedness is low), irrespective of ${\it{\epsilon }}$ (Fig. 2e). At high clonality (1−${\it{\epsilon }}$) and high non-clonal inoculum size (*N*), allele diversity of facultative cooperation is therefore maintained concomitantly with being stable against cheating (Outlined area in Fig. 2d, e). In the case of partial migration, fitness is determined by both growth (fecundity) and competition within a patch [39] and population structure becomes more complex as well. This, however, does not qualitatively affect our results (see SI text, and Fig. S1). The separation into two types of patches is conceptually simple, but not necessary. The same qualitative effects are found when we consider the case where a fraction (1−${\it{\epsilon }}$) is clonal while the rest of the microbes inoculate patches with a varying (long-tailed) distribution of patch sizes (SI text, Fig. S2).

**Fig. 3:**
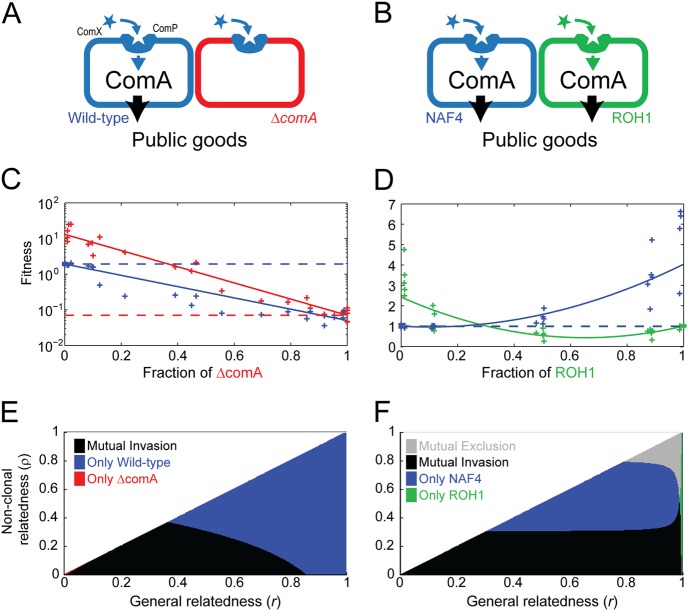
Analysis of experimental data of two social interactions between quorum-sensing variants in *B. subtilis*. **a**, **c**, **e** Interaction between the wild-type cooperator strain (in blue), which produces and secretes Surfactin (a public-good surfactant that enables swarming motility) and a non-producing cheater mutant (in red). Surfactin is produced in response to the ComQXP QS system. ComX is a signaling peptide that binds and activates the receptor ComP, which then activates downstream response via ComA. When co-cultured in a swarming assay, a strain deleted of the response regulator gene *comA* (in red), exploits the production of Surfactin by the wild-type strain and swarms well, leading to a fitness advantage of the mutant over the wild-type in any mixed culture. **b**, **d**, **f** Interaction between isogenic *B. subtilis* strains encoding for pherotypes (receptor-signal allele pairs) RO-H-1 (in green) and NAF-4 (in blue) of the ComQXP QS system. A strain of each pherotype responds specifically to its own signaling peptide and produces Surfactin proportionally to its frequency in co-culture, leading to mutual facultative cooperation. **a–b** Social interaction schemes. **c**–**d** Frequency-dependent fitness values (plus signs, data taken from ref. [16]) with least squares fits (solid lines, see Methods) and dashed lines that emphasize clonal fitness values. **e**–**f** Calculated invasion plots in a simple structured population, where general and non-clonal relatedness vary continuously and independently (although *ρ* is always smaller than *r*) (Methods). Colored areas report the success of invasion of each of the variants, when rare

#### Colony expansion model

Microorganisms also achieve high clonality via colony or biofilm growth. It was previously shown that under such growth conditions, the combination of genetic drift and competition for space and nutrients leads to the formation of clonal “sectors”, even if competing genotypes are neutral [28, 40]. Selection will affect both the probability of sector emergence and the growth of emerging sectors [41]. Selection for cooperative behaviors under such conditions has been demonstrated both theoretically [30] and experimentally [27], but little attention has been given to the fate of kin-discriminative strategies under these modes of growth.

We used lattice-based, two-dimensional simulations to analyze the emerging selection patterns during colony growth [42]. We simulated colony growth on a two-dimensional lattice, where each lattice point is a microbe (Fig. 2f) [43]. Microbes were initially arranged in a disc of a ~20-cell radius. We assumed that only microbes on the colony border are socially active and able to grow. At the start of a simulation, *N*_*i*_ random nearby microbes on the border were chosen to be of the rare genotype, while the rest were of the resident genotype. At each time step, each active microbe was assigned a fecundity value based on its genotype, the applied social rules (Fig. 2a, b) and the frequency *G* of invaders within the active population in a squared (*n* × *n*) social neighborhood centered around it (Fig. 2f). A single microbe was then randomly chosen at a probability proportional to its fecundity and a progeny of the same genotype was placed in an adjacent open lattice position. The simulation then continued to the next time step.

Under the above assumptions, the fate of a given genotype is determined by its social nature, social neighborhood, local competition and random drift in a complex manner. In any simulation instance, a sector of the rare genotype may form (Fig. 2f). Both the probability of sector formation and its growth will depend on the different parameters, in a stochastic manner. We therefore ran ~80,000 repeats of the simulation and followed the frequency of the invader in the meta-population composed of all simulation repeats, for an order of 10^5^ time steps per run.

We first considered the case of a single initiating microbe of the rare genotype (*N*_*i* _= 1). We found that the average frequency of a rare cheater initially increased, but this trend was subsequently reversed (Fig. 2g). Facultative-cooperator invasion into a population of cheaters exhibited the opposite trend, with an initial decrease, followed by a subsequent increase in frequency (Fig. S3). Finally, the frequency of the facultative cooperator, invading another facultative cooperator, initially increased similarly to the cheater genotype, but then remained nearly constant with a very slow decline (Fig. 2g). By the end of the simulation run, these trends resulted in the invasion of facultative cooperators into the cheater and into another facultative cooperator, while the rare cheater failed to invade (Fig. 2g, Fig. S3).

The above results can be qualitatively understood by considering selection on the clonal and non-clonal subpopulations in each type of social interaction. To better understand this, we calculated the three population-structure measures, *r*, *ρ* and, *x*_1*c*_ for the meta-population of all simulation repeats (Fig. 2h). Each of these variables was calculated based on the social neighborhood defined above (Fig. 2f). We found that these variables behave similarly for all types of interactions (Fig. S3), so for simplicity, we present here those of the facultative cooperator invading another facultative cooperator (Fig. 2h). Initially, both measures of relatedness were low ($r = \rho \approx \frac{{N_i}}{n}$) and the invader resided in non-clonal groups only. At later simulation steps, clonal sectors appeared and clonality level increased. This was concurrent with a marked increase in general and non-clonal relatedness. The change in population structure measures over the course of the simulation correlated well with the initial selection and later counter-selection of invading cheaters and the opposite behavior of invading facultative cooperators. For the interaction between the two facultative-cooperation variants, initial invasion was well correlated with the initial low non-clonal relatedness. At later times, the facultative-cooperator’s extremely weak counter-selection corresponded well with the increase in non-clonal relatedness and the reduced fraction of non-clonal groups, which solely dictated selection.

The dynamics of colony growth invasion can be partitioned into early (non-clonal) and late (predominantly clonal) phases, whose integration determines evolutionary fate. The facultative-cooperator’s invasion into another facultative cooperator is expected to depend mostly on the dynamics in the early, non-clonal phase, while the cheater invasion is expected to depend on both phases. We therefore inspected the significance of these phases by varying both the initial relatedness (by changing *N*_*i*_), and the duration of the late phase (by varying the overall number of time steps, *T*). Indeed, invasion of the facultative cooperator into another facultative cooperator was only weakly dependent on the duration of the late phase, but strongly depended on initial relatedness (invasion succeeds for *N*_*i*_, ≤ 3), while cheater invasion depended on both parameters (Fig. 2i, j). Initial relatedness and the duration of the predominantly clonal phase therefore play a similar role to the inverse bottleneck size, $\frac{1}{N}$, and clonality level, 1−${\it{\epsilon }}$, in the infinite island model, and facultative cooperators coexist and resist cheating in a range of parameters (Outlined area in Fig. 2i, j). In addition, we found that the invasion pattern did not qualitatively depend on social neighborhood size (*n*), or the initial colony size, but did depend in an expected manner on the cost to benefit ratio (Fig. S3).

### Population structure and the evolution of quorum-sensing systems

Our theoretical analysis provides a means to predict the effect of population structure on kin-discriminative interactions. We recently showed experimentally that the ComQXP quorum-sensing (QS) system of the Gram-positive bacterium, *Bacillus subtilis*, controls cooperative behaviors and that a QS response mutant behaves as a social cheater in a swarming motility assay by exploiting the wild-type’s production of a public-good surfactant (Fig. 3a, c). We further showed the occurrence of mutual facultative cooperation between divergent QS alleles (also known as pherotypes) (Fig. 3b, d) [16, 44]. Each pherotype senses only its own signal. A majority pherotype therefore senses stronger signal and correspondingly makes more surfactant than the minority strain, leading to a fitness advantage of the minority pherotype. In the best-studied case (comparison of pherotypes NAF4 and RO-H-1) sensing levels were not equal, which led to a non-symmetric coexistence point (with a ratio of ~4:1), but equal clonal fitness (Fig. 3d). Notably, some *Bacillus* pherotypes show a low level of cross-interaction, while others show negative cross-talk [45, 46], but this does not qualitatively changes the existence of mutual facultative cooperation [21, 46].

We used the data collected in the above experiments to analyze the selection on realistic kin-discriminative and exploitive behaviors in different population structures. To this aim, we considered the extreme bottleneck scenario, treating each swarm as the output of a single growth patch (*Bacillus subtilis* swarms have been shown to be well-mixed and unstructured [16]). We transformed the data presented in our previous work [16], which showed total cell yield and relative frequency changes of the two genotypes, to the fitness of each genotype as a function of genotype frequencies (Fig. 3c, d, Methods). We then used fitted functional forms of the fitness to calculate the ability of each of the genotypes to invade when rare, in a simple structured population, similar to the infinite island model described above, where *r* and *ρ* can be varied independently and continuously (Fig. 3e, f, Methods). In such a model, selection of a rare variant is established by comparing its fitness over the distribution of subgroups it resides in, to the clonal fitness of the resident variant. Note that the strong non-linearity of the fitness functions may lead to different relatedness thresholds for invasion when the rare and the resident genotypes are switched, allowing for mutual invasion or mutual exclusion of two genotypes.

We found that selection in these realistic social interactions followed a similar scheme to that obtained with simplistic assumptions on the nature of social interactions. First, we examined the interaction between the *∆comA* QS cheater mutant [16] and the wild-type QS cooperator (Fig. 3a, c, e). At low levels of general and non-clonal relatedness, the cheater and cooperator co-existed by mutual invasion. As general or non-clonal relatedness increased, the cheater lost its ability to invade. Cheater dominance occurred near zero relatedness only. Clearly, cheating dynamics depends strongly on both measures of population structure (Fig. 3e).

Next, we examined the interaction between the two QS pherotypes named RO-H-1 and NAF-4 (Fig. 3b, d, f) [16]. The minimal fitness of both strains in well-mixed swarming plates occurred at an intermediate frequency (Fig. 3d), suggesting that selection will depend on population structure (Box Fig. B). This is reflected in the invasion plot of the two strains (Fig. 3f). Except for at very high general relatedness (*r* < 0.95), invasion depended predominantly on non-clonal relatedness. Each of the strains invaded below a certain threshold of non-clonal relatedness (*ρ* < 0.3 for the RO-H-1, and *ρ* < 0.8 for NAF-4) corresponding to their asymmetric coexistence point in well-mixed swarming plates. At the high *r* region, invasion was dependent on both general and non-clonal relatedness, although experimental accuracy was insufficient to determine it (Fig. S4). This dependence on the general relatedness in the high *r* region is the result of the small, non-significant, difference in clonal fitness of the two variants, which leads to dominance of the clonal component in Eq. 1. These results suggest that, for realistic interactions as well, facultative-cooperation allele diversity could be both maintained and stabilized against cheating under high clonality and low non-clonal relatedness.

## Discussion

In this work, we analyzed the impact of population structure on diversity of kin-discrimination alleles and demonstrated that clonality impacts the balance between kin-discriminative interactions and other social interactions. Specifically, we showed that high clonality, combined with mixing in the non-clonal part of the population, maintains facultative-cooperation allelic diversity and stabilizes it against the invasion of cheaters. The intuition for this effect is simple (Fig. 1) – clonal groups promote cooperation, but do not have any effect on interactions between facultative-cooperation variants.

Using the Price equation, we devised a modified version of Hamilton’s rule that demonstrates that the fate of an invading kin-discrimination genotype is not dependent on the clonal component of the general relatedness coefficient but it may depend on a novel measure we defined. This coefficient, termed non-clonal relatedness, measures identity of interacting organisms in genetically heterogeneous social groups, whereas general relatedness measures identity over all social groups, including clonal ones (Box). We stress that the theoretical analysis we performed does not diverge from the framework of the Price equation and Hamilton’s rule [33].

Our analysis points to the importance of non-linear frequency dependence when considering kin-discriminative interactions. In fact, we showed that linear fitness functions would necessary lead to a population-structure-independent invasion pattern. From a mechanistic perspective, strong non-linear frequency dependence characterizes many microbial social interactions, which typically depend on public goods [13, 47, 48]. Specifically, facultative cooperation is intrinsically non-linear, as the level of cooperation depends on the frequency of cooperating cells in a population. By re-analyzing experimental data we have recently published, we showed how the realistic interaction between QS pherotypes [16] displays approximately equal clonal fitness and non-linear frequency dependence, with a minimum fitness at an intermediate frequency. This allows for the diversifying selection of QS-mediated facultative cooperation under certain structured populations (Fig. 3). The robustness of the results to differences in the clonal fitness of the two QS pherotypes suggests the generalization of our predictions to cases where kin-discrimination variants also differ in clonal fitness but this difference is much smaller than fitness effects in mixed cultures.

While Hamilton’s rule is always true, it is not always resourceful in understanding the dependence of selection on the parameters of specific models [33, 49]. We therefore studied the relevance of our insights on facultative cooperation in two specific microbial growth models. In these models, kin-discriminative interactions were only assumed for the growth (fecundity) component of the life cycle, while reproductive success of a given organism was also dictated by other aspects of the cycle, such as migration, local competition and chance. In both growth models, we were able to show how the different parameters relate to the theory and how one parameter (${\it{\epsilon }}$ in the islands model, and the number of simulation steps *T* in the colony growth model) strongly affects the stability of cooperation against cheaters due to its impact on clonality, while not significantly affecting interactions between two facultative cooperators (Fig. 2). This led to the maintenance of facultative-cooperation allele diversity together with stability against cheaters in a wide range of parameters. Especially important is the colony growth model, since in this model clonality is not externally invoked (as in the infinite island model), but is an emergent property of the growth process, as has been shown by many theoretical and experimental works [27, 28, 30, 40, 41]. The direct relevance of this model to experimental systems, also allows one to envision relevant experiments, where biofilms could be grown under varying external conditions (which affect sector formation [41]), or initial population size (which affects initial mixing [50]), to test the effect of clonality and mixing on kin-discriminative compared to other social interactions, and specifically on the maintenance of facultative-cooperation allele diversity. From a modeling perspective, there are multiple ways of simulating social interactions on grids, by differently connecting a fitness parameter to growth laws or implicating the effect of the social neighborhood [32, 51]. While we chose a specific set of rules for our simulation, we expect that modification of these rules will not considerably change the result, but such comparison will be done elsewhere.

What is the impact of clonality on the evolution of other kin-discriminative interactions? The answer to this question depends on the specific mechanism of kin discrimination and on the range of possible additional social interactions that would impact the system upon recombination or mutation. In the SI text, we consider another example of a kin-discriminative interaction (in our broad definition), between a facultative-harming “greenbeard” and a non-beard organism [52]. We show that both high clonality and high non-clonal relatedness are needed to maintain the dominance of the greenbeard over a nonbeard, if the non-beard can acquire a costly resistance to the harming effect (SI text and Fig. S5).

Much of the literature on diversity of kin-recognition alleles is focused on kin-directed help and non-kin directed harm which lead to positive frequency-dependent selection between divergent alleles. A possible explanation for the maintenance of diversity in such systems depends on oscillations in dominance between different kin-discrimination cooperating genotypes and their respective cheaters [1, 2, 34, 52–60]. Our analysis points to an additional possible mechanism, where positive frequency dependence is reversed into negative frequency dependence between strains if non-clonal relatedness is high enough. This would only be possible if frequency dependence is non-linear and the maximum of the invader’s fitness is obtained at an intermediate frequency (Box Fig.). Although such non-monotonic frequency dependence is harder to imagine considering growth (fecundity) only, local competition might generate it at the fitness level. We demonstrate this theoretical prediction in a colony growth simulation (Fig. S6).

Kin-discrimination also evolves by combinatorial accumulation of multiple different kin-discrimination loci in the same genome. In *B. subtilis* this was shown to occur both for toxin-antidote (non-kin harm) loci [61, 62] and for quorum-sensing systems of the Rap-Phr type [17, 63]. Understanding the accumulation of a kin-discrimination locus requires studying its interaction with an organism lacking it. If the novel locus has low cost in clonal groups, this will lead to a kin-discriminative interaction. We show in the SI text and Fig. S7 that in this case, the invasion of the strain with the additional locus will always be favored, both for non-kin harm and for facultative cooperation.

Discriminating mechanisms can also work at the species level. For example, recent works showed how diversity of QS signals between vibrio species may lead to facultative-cooperation between species [17, 64]. We note that the mechanisms we proposed could also stabilize ecological coexistence of related species which tend to overlap in their niches and can exploit each other’s production of public goods.

Altogether, our work points to the importance of microbial clonal structure and non-linear interactions in the evolution of various kin-discriminative interactions. While characterization of social interactions by simple measures like benefit, cost and relatedness often masks much of the complexity of social interactions, it also allows for broad generalizations and the ability to (carefully) draw analogies between distinct social systems [33]. Here, we showed how such simplification enables drawing some general conclusions regarding the complex social phenomena of microbial facultative cooperation.

## Methods

### Experimental data analysis

For each of the two interactions (Fig. 3a, b), we used total co-culture yield, as well as initial and final fractions of the two strains in co-culture, presented in [16]) (in the pherotypes dataset (Fig. 3d), two technical repeats were averaged to present one biological repeat). To reduce the impact of day-to-day variation in yield, we normalized the total co-culture yield in each of the five biological repeats of each dataset by the mean yield in that repeat. The fitness *W* of each strain was obtained by multiplying the normalized yield by the ratio of final to initial fractions of that strain (Fig. 3c, d).

Fitness of each strain as a function of its fraction, was fitted with a weighted fit, using the fit command in MATLAB Curve Fitting Toolbox R2015b (MathWorks, Inc., Natick, Massachusetts) (Fig. 3c, d). A linear fit in log scale was used in Fig. 3c, and a parabolic fit in linear scale was used in Fig. 3d, where fitness was non-monotonic. Datasets were binned according to initial fractions, with bins of ~1, ~10, ~50, ~90, ~99, and 100%. The fitting weight in each bin was calculated as the inverse of the variance in that bin.

For population structure, we extended the dual-bottleneck infinite island model, presented above, to allow continuous variation in general and non-clonal relatedness coefficients. This population structure can be presented by the following three-parameter distributions of social neighborhoods:

4 $$\begin{array}{l}P\left( G \right) = \delta \left( G \right)\left( {1 - p_1\frac{{1 - r + \rho }}{\rho }} \right) + \delta \left( {G - \rho } \right)p_1\frac{{1 - r}}{{\rho \left( {1 - \rho } \right)}}\\ + \, \delta \left( {G - 1} \right)p_1\frac{{r - \rho }}{{1 - \rho }}\end{array}$$

where *p*_1_,*r*,*ρ* are parameters that satisfy 0 < *ρ* ≤ *r* < 1 and *p*_1_→0 (for invasion from rarity). Therefore, individuals populate clonal groups of genotype #2 at a probability $P\left( {G = 0} \right) = 1 - p_1\frac{{1 - r + \rho }}{\rho }$, mixed groups with a fraction *ρ* of genotype #1 individuals at a probability $P\left( {G = \rho } \right) = p_1\frac{{1 - r}}{{\rho \left( {1 - \rho } \right)}}$, and clonal groups of genotype #1 at a probability $P\left( {G = 1} \right) = p_1\frac{{r - \rho}}{{1- \rho}}$. This distribution yields: 〈*g*〉 = *p*_1_, *r* = *r*, and *ρ* = *ρ* for the invader frequency, general and non-clonal relatedness coefficients, respectively.

Using Eq. S8 (SI text), we calculated the conditions for invasion in this population structure (presented in Fig. 3e, f):

5 $$\frac{{1 - r}}{{1 - p}}W_1\left( \rho \right) + \frac{{r - \rho }}{{1 - \rho }}W_1\left( 1 \right) - W_2\left( 0 \right)\hskip 2pt > \hskip 2pt 0.$$

where we used the fitting functions in order to estimate the above values of the fitness function.

## Electronic supplementary material

Supplementary text

Table S1

Table S2

Table S3

Table S4

Table S5

Table S6

Figure S1

Figure S2

Figure S3

Figure S4

Figure S5

Figure S6

Figure S7
