# Supplementary material for: Clonality and non-linearity drive facultative-cooperation allele diversity
Source: ISME J. 2018 Nov 21;13(3):824–35. doi: 10.1038/s41396-018-0310-y (PMC6461992; doi:10.1038/s41396-018-0310-y)
Supplement: Supplementary file 1 — Supplementary text [file 41396_2018_310_MOESM1_ESM.docx]

**Supplementary information**

**Clonality and non-linearity drive facultative-cooperation allele diversity**

Ishay Ben-Zion, Shaul Pollak & Avigdor Eldar

**Contents**

[Supplementary text 2](#_Toc529312063)

[Model formulation 2](#_Toc529312064)

[General and non-clonal relatedness 4](#_Toc529312065)

[Dependence of invasion on the shape of the fitness function 4](#_Toc529312066)

[Non-monotonicity of facultative-cooperation frequency dependence 5](#_Toc529312067)

[Infinite island model with migration 7](#_Toc529312068)

[Life cycle 7](#_Toc529312069)

[Analysis method 8](#_Toc529312070)

[Calculating the stationary distribution of patch composition under neutrality 8](#_Toc529312071)

[Using the stationary patch distribution in the Price equation 10](#_Toc529312072)

[Analytical derivation of the invasion conditions for mutual facultative cooperation and exploitive interactions 12](#_Toc529312073)

[Infinite-island model with a distribution of patch sizes 14](#_Toc529312074)

[The impact of clonality on the evolution of resistance to a facultative-harming greenbeard 15](#_Toc529312075)

[Error estimation for experimental data analysis 17](#_Toc529312076)

[Accumulation of kin-discrimination loci 18](#_Toc529312077)

[Supplementary text references 20](#_Toc529312078)

[Supplementary tables 23](#_Toc529312079)

[Supplementary figures 26](#_Toc529312080)

# Supplementary text

## Model formulation

We considered a neighbor-modulated representation of the social interaction in a structured population between two genotypes; genotype $\#1$ with genetic value $g=1$, and genotype $\#2$ with genetic value $g=0$, with no mutation, no recombination and no class structure. The social environment of a focal individual is measured by $G$, defined as the frequency of genotype $\#1$ in the social neighborhood of the focal individual (including self). The relative fitness $W$ of an individual (defined as the growth factor of its lineage during a time step, divided by the mean growth factor) is dictated by its genetic value $g$, by its social environment $G$, and by other environmental parameters denoted by $E$; $W=W\left( g,G,E \right)$. The fitness can be written as $W\left( g,G,E \right)=gW_{1}\left( G,E \right)+\left( 1-g \right)W_{2}\left( G,E \right)$ where $W_{i}\left( G,E \right), i=1,2$ is the fitness function of an individual of genotype $\#i$.

For any given population structure with a defined $P\left( G \right)$ – the distribution of social neighborhoods of individuals in the population, we can use the Price equation [1, 2] to calculate the change in the overall frequency of genotype $\#1$ during a time step:

1. $\Delta p_{1}=cov\left( W,g \right)=\left\langle Wg \right\rangle-\left\langle W \right\rangle p_{1}$,

where $p_{1}\equiv\left\langle g \right\rangle$, is the frequency of genotype #1 in the population and brackets $\left\langle\right\rangle$ with no subscript denote averages done on all individuals in the population. Hamilton’s rule is obtained by replacing the regression coefficient of fitness on genetic value, $\beta_{Wg}=\frac{cov\left( W,g \right)}{var\left( g \right)}$, by the two-dimensional linear regression on $g$ and $G$:

1. $\beta_{Wg}=\beta_{\left. WG \right|g}\beta_{Gg}+\beta_{\left. Wg \right|G}$,

where $\beta_{\left. WG \right|g}\equiv b$ and $\beta_{\left. Wg \right|G}\equiv-c$ measure the two ways the individual’s genes affect its fitness (directly, and through copies of these genes in its social partners), and $\beta_{Gg}\equiv r$ is Hamilton’s relatedness coefficient [2]. Hamilton’s condition for the increase in the frequency of genotype $\#1$ is therefore:

1. $rb-c>0$.

We can now rewrite this condition by dividing all averages to two sub populations. One subpopulation, which we denote with the subscript $1c$, stands for the subpopulation of individuals of genotype $\#1$ that are in clonal interaction groups $\left( G=1 \right)$. The frequency of this sub-population in the general population is denoted by $p_{1c}\equiv p_{1}x_{1c}$ (where $x_{1c}$, which we term the clonality level, is the frequency of this sub population among organisms of genotype $\#1$). The second subpopulation, which is denoted with the subscript $nc$, stands for the remaining (non-clonal) subpopulation that includes genotype #1 organisms which are in non-clonal social groups and all genotype #2 organisms. Each average over the whole population could be divided according to: $\left\langle\right\rangle=p_{1c}\left\langle\right\rangle_{1c}+\left( 1-p_{1c} \right)\left\langle\right\rangle_{nc}$, where brackets with subscript denote averages done on the relevant subpopulation. Using the fact that $g=1$ in the clonal subpopulation, and therefore $\left\langle g \right\rangle_{1c}=1$ and $\left\langle Wg \right\rangle_{1c}=\left\langle W \right\rangle_{1c}$, we find that:

1. $cov\left( W,g \right)=p_{1c}\left( 1-p_{1c} \right)\left( \left\langle W \right\rangle_{1c}-\left\langle W \right\rangle_{nc} \right)\left( 1-\left\langle g \right\rangle_{nc} \right)+\left( 1-p_{1c} \right)cov_{nc}\left( W,g \right)$.

We proceed with the same two-dimensional regression as done in Hamilton's rule (Eq. S2), but only on the non-clonal subpopulation:

1. $cov_{nc}\left( W,g \right)=var_{nc}\left( g \right)\left( \beta_{WG|g}^{nc}\beta_{Gg}^{nc}+\beta_{Wg|G}^{nc} \right)=\left\langle g \right\rangle_{nc}\left( 1-\left\langle g \right\rangle_{nc} \right)\left( \rho b_{nc}-c_{nc} \right)$,

where we denote the relatedness coefficient on the non-clonal subpopulation as $\rho\equiv r_{nc}$. Using the relation $p_{1}=p_{1c}+\left( 1-p_{1c} \right)\left\langle g \right\rangle_{nc}$, we get:

1. $\frac{\Delta p_{1}}{p_{1}\left( 1-p_{1} \right)}=x_{1c}\left( \left\langle W \right\rangle_{1c}-\left\langle W \right\rangle_{nc} \right)+\frac{1-x_{1c}}{1-p_{1}x_{1c}}\left( \rho b_{nc}-c_{nc} \right)$.

This can be further modified to the following form:

1. $\frac{\Delta p_{1}}{p_{1}\left( 1-p_{1} \right)}=x_{1c}\left( \left\langle W \right\rangle_{1c}-\left\langle W \right\rangle_{2} \right)+\left( 1-x_{1c} \right)\left( \rho b_{nc}-c_{nc} \right)$.

Here, the $\left\langle\right\rangle_{2}$ notation means averaging over the second genotype's population only. In the limit where genotype $\#1$ is rare, we have: $\left\langle W \right\rangle_{2}=\left\langle W \right\rangle_{2c}+O\left( p_{1} \right)$, where $\left\langle W \right\rangle_{2c}$ is the mean fitness over clonal groups of genotype $\#2$, and this leads to the version of Hamilton’s rule for an invasion scenario, presented as Eq. 1 in the main text:

1. $\frac{\Delta p_{1}}{p_{1}}=x_{1c}\left( \left\langle W \right\rangle_{1c}-\left\langle W \right\rangle_{2c} \right)+\left( 1-x_{1c} \right)\left( \rho b_{nc}-c_{nc} \right)+O\left( p_{1} \right)$.

For kin-discriminative interactions, we get:

1. $\frac{\Delta p_{1}}{p_{1}}=\left( 1-x_{1c} \right)\left( \rho b_{nc}-c_{nc} \right)+O\left( p_{1} \right)$.

This is translated to the invasion condition for kin-discriminative interactions, presented in Eq. 2 in the main text.

## General and non-clonal relatedness

To calculate general and non-clonal relatedness, we used the following formula for relatedness [3], which separates averages to the two subpopulations of the two genotypes:

1. $r=\frac{Cov\left( G,g \right)}{var\left( g \right)}=\left\langle G \right\rangle_{1}-\left\langle G \right\rangle_{2}$.

In the limit where genotype $\#1$ is rare, we have $r=\left\langle G \right\rangle_{1}+O\left( p_{1} \right)$. In the same way we separate the non-clonal subpopulation and obtain non-clonal relatedness:

1. $\rho=\left\langle G \right\rangle_{1,nc}-\left\langle G \right\rangle_{2}=\left\langle G \right\rangle_{1,nc}+O\left( p_{1} \right)$.

The relation between these two relatedness measures (Eq. 3 in the main text), in this limit, is therefore:

1. $r=x_{1c}+\left( 1-x_{1c} \right)\rho$.

## Dependence of invasion on the shape of the fitness function

Another way to separate the covariance in Eq. S1 is by averaging over the two genotypes separately:

1. $\frac{\Delta p_{1}}{p_{1}\left( 1-p_{1} \right)}=\frac{cov\left( W,g \right)}{p_{1}\left( 1-p_{1} \right)}=\left\langle W \right\rangle_{1}-\left\langle W \right\rangle_{2}$,

where $\left\langle\right\rangle_{i}$ denotes averaging over the subpopulation of genotype $\#i$ only. The same could be done in the non-clonal subpopulation (for kin-discriminative interactions), in the limit where genotype $\#1$ is rare (Eq. S9):

1. $\frac{\Delta p_{1}}{p_{1}}=\left( 1-x_{1c} \right)\left( \left\langle W \right\rangle_{1,nc}-\left\langle W \right\rangle_{2c} \right)+O\left( p_{1} \right)$

$$=\left( 1-x_{1c} \right)\left( \left\langle W \right\rangle_{1,nc}-\left\langle W \right\rangle_{1c} \right)+O\left( p_{1} \right)$$

where $\left\langle W \right\rangle_{1,nc}$ is the average fitness of genotype $\#1$ individuals in the non-clonal subpopulation and we used the fact that $\left\langle W \right\rangle_{2}=\left\langle W \right\rangle_{2c}+O\left( p_{1} \right)$ when genotype #1 is rare and $\left\langle W \right\rangle_{2c}=\left\langle W \right\rangle_{1c}$ for kin-discriminative interactions.

If we now further assume that the invader’s fitness, $W_{1}\left( G,E \right)$, is continuous and depends only on $G$ we find that if $W_{1}\left( 1 \right)$ is a global minimum of the fitness function, then $\left\langle W \right\rangle_{1,nc}>W_{1}\left( 1 \right)=\left\langle W \right\rangle_{1c}$. This implies that invasion will succeed, irrespective of the population structure, $P\left( G \right)$. Clearly, the opposite direction is also correct. If $W_{1}\left( 1 \right)$ is not a global minimum, then there would exist a distribution $P(G)$ over which the average would be smaller than $W_{1}\left( 1 \right)$ and invasion would fail. Similar arguments hold if $W_{1}\left( 1 \right)$ is (or is not) a global maximum.

## Non-monotonicity of facultative-cooperation frequency dependence

In the main text, we argue that two interacting facultative cooperators are likely to display non-monotonous frequency dependent selection with a fitness minimum at an intermediate frequency and not at clonality (i.e., frequency of 1). To show that, we assume two symmetric facultative cooperators with different kin-discrimination alleles that provide the cell information on the frequency of kin in the population. We designate by $G$ the frequency of genotype #1 and $1-G$ the frequency of genotype #2. We assume that each cell is producing a public good $X$, as a function of its genotype frequency $p$, and this carries a cost $C\left( X\left( p \right) \right)$. The benefit to the cell is a function of the average public-good level in the population, $B\left( \left\langle X \right\rangle\right)$. The fitness of each cell is assumed to be a basal term plus the benefit minus the cost. For genotype #1 this implies:

1. $W_{1}\left( G \right)=W_{0}+B\left( GX\left( G \right)+\left( 1-G \right)X\left( 1-G \right) \right)-C\left( X\left( G \right) \right)$.

Using Taylor expansion, we find the expression for the fitness at a frequency approaching one, $G=1-\epsilon$:

1. $W_{1}\left( 1-\epsilon\right)\approx W_{1}\left( 1 \right)-\epsilon\left( \underset{term 1}{\underbrace{\left( X\left( 1 \right)-X\left( \epsilon\right) \right)B^{'}\left( X\left( 1 \right) \right)}}+X^{'}\left( 1 \right)\underset{term 2}{\underbrace{\left( B^{'}\left( X\left( 1 \right) \right)-C^{'}\left( X\left( 1 \right) \right) \right)}} \right)$

The first term of the correction is the net change in benefit due to the reduction of majority genotype frequency and increase in minority genotype frequency. The second term is due to the change in public-good investment by the majority cooperator with its reduced frequency. If we assume that public-good production, benefit and cost are all monotonously increasing functions than the first term is immediately positive. The second term is the derivative of the fitness in clonal populations with respect to changes in public-good level. This term may either be positive or negative, but is close to zero, assuming that public-good production is close to its optimal level (i.e., the fitness derivative is close to zero). The total correction term for the fitness is therefore negative under reasonable assumptions. This implies that clonal fitness is not minimal.

To analyze the effect of non-clonal relatedness on coexistence of facultative cooperators, we assume for simplicity that the invader's fitness has only one local minimum (e.g., Box 1 Fig. B in the main text). Since the fitness of a minority facultative cooperator is higher than one (the clonal fitness), this would also be true for any frequency $G$ below some point $G_{0}$, while the fitness at $G>G_{0}$ would be lower than one. Non-clonal relatedness $\rho$ is the mean of $G$ over invaders in non-clonal groups (Eq. S11), thus when $\rho$ is low enough, most of the invaders will experience $G$ that is lower than $G_{0}$, and the mean invader fitness will be higher than one. On the other hand, if $\rho$ is high enough most invaders will experience $G>G_{0}$ and the mean invader fitness will be lower than one. This implies that coexistence of facultative cooperators would only be possible when non-clonal relatedness is low enough.

## Infinite island model with migration

### Life cycle

We defined the infinite island model similar to previous other works (e.g., [4-8]), with a few changes. As in the general model above, we assumed that one genotype ($i=1$) is in a very small fraction $p_{1}$ in a population of a resident genotype ($i=2$). Second, we assumed that an infinite population is distributed to a number of $I\to\infty$ patches of two sizes. A fraction $\epsilon$ of individuals inoculates large patches, formed by $N$ independent individuals, and the rest ($1-\epsilon$) inoculate small patches formed by a single individual. Third, we assumed that all patches first grow by a factor of $M$ (as in [8, 9]), and only then start to interact socially (also known as interaction between juveniles [8] or lineages [10]). The fecundity at this stage of a lineage (that started from one organism in the original patch) of genotype $i$ in a patch with a fraction $\frac{j}{N}$ of invaders is proportional to: $1+f_{i}\left( \frac{j}{N} \right)$, where we assumed that $f_{2}\left( 0 \right)=0$ to get mean population fitness equal to 1. The social growth rules that define the fecundity are given below (and in Fig. 2A,B in the main text). After social growth, each microbe either migrates, at a probability $m$, to a randomly chosen patch (where the probability of choosing a patch is proportional to its inoculum size), or stays in its patch at a probability $1-m$. Finally, a new cycle arises by random sampling of each patch population back to its original inoculum size (either 1 or $N$). We assumed that $f_{i}\left( \frac{j}{N} \right)$ are much smaller than $m$ to work in the weak selection regime. As in similar models, we assumed that the growth stages occur instantaneously (each microbe produces a large number of offspring at once and dies) so drift is negligible [8, 11, 12].

### Analysis method

We present here a patch distribution analysis method for calculating selection in an infinite island model, similar to that presented by Gardner & West [8], which is different than the coalescence approach used more often [11]. This approach is better fit for the analysis of non-linear fecundity functions. We first derived the stationary distribution of patch composition under neutrality (valid in the first order approximation of the weak selection regime [5], since the patch distribution equilibrates much faster than the change in frequency $p_{1}$). We then utilized this distribution to calculate selection.

### Calculating the stationary distribution of patch composition under neutrality

We assume that the two genotypes are neutral and calculate the stationary distribution of patch composition. We can calculate the stationary distributions for the two patch sizes independently, because the two subpopulations are infinite so $p_{1}$ is the invader fraction in each of them. The clonal patches simply distribute to fractions $p_{1}$ and $1-p_{1}$ for the invader and resident patches, and this is also the stationary distribution under neutrality.

#### Non-clonal patches

For the non-clonal patches, we define $P_{Nj}\left( k \right)$as the probability that a given patch of size $N$ will have $j$ founders of genotype $\#1$ at cycle $k$. The total frequency of genotype $\#1$ is therefore:

1. $p_{1}=\sum_{j=1}^{N} \frac{j}{N}P_{Nj}\left( k \right)$.

Under the limit where genotype #1 is rare, we can take only terms of the first relevant order in $p_{1}$. The life cycle described above implies that the distribution of patches in the next stage, under neutrality, will be (for $i=1\ldots N$):

1. $P_{Ni}\left( k+1 \right)=\sum_{j=0}^{N} \left( \begin{aligned} N \\ i \end{aligned} \right)\left( {\tau^{'}}_{j} \right)^{i}\left( 1-{\tau^{'}}_{j} \right)^{N-i}P_{Nj}\left( k \right)$

where $\tau_{j}^{'}=\left( 1-m \right)\frac{j}{N}+mp_{1}$ is the mean fraction of genotype $\#1$ individuals in a patch of previous type $j$, after growth and migration but before sampling. Since $M$ is assumed to be large, we can neglect stochasticity in migration, i.e., we can assume that for each original individual the fraction of its offspring that migrate is exactly $m$.

This can be further simplified to:

1. $P_{Ni}\left( k+1 \right)=mNp_{1}\delta_{i1}+\sum_{j=1}^{N} \left( \begin{aligned} N \\ i \end{aligned} \right)\left( \left( 1-m \right)\frac{j}{N} \right)^{i}\left( 1-\left( 1-m \right)\frac{j}{N} \right)^{N-i}P_{Nj}\left( k \right)+O\left( p_{1}^{2} \right)$,

where $\delta_{i1}$is Kronecker delta. The stationary solution is defined by the equation:

1. $\tilde{P}_{Ni}\left( k+1 \right)=\tilde{P}_{Ni}\left( k \right)=\tilde{P}_{Nj}$.

We can define the vector ${\tilde{\boldsymbol{\Pi}}}_{\boldsymbol{N}}\boldsymbol{\in}\mathbb{R}^{\boldsymbol{N}}$ as $\tilde{\Pi}_{Nj}=\frac{\tilde{P}_{Nj}}{p_{1}}, j=1,\ldots,N$, and write Eq. S19 in the stationary case in a vector form:

1. ${\tilde{\boldsymbol{\Pi}}}_{\boldsymbol{N}}\left( k+1 \right)=\boldsymbol{A}{\tilde{\boldsymbol{\Pi}}}_{\boldsymbol{N}}\left( k \right)+\boldsymbol{v}$,

with the matrix $\boldsymbol{A}$ and vector $\boldsymbol{v}$ defined by, $A_{ij}=\left( \begin{aligned} N \\ i \end{aligned} \right)\left( \left( 1-m \right)\frac{j}{N} \right)^{i}\left( 1-\left( 1-m \right)\frac{j}{N} \right)^{N-i}$, and $v_{i}=\delta_{i1}mN$. The stationary distribution of patches therefore follows the equation:

1. ${\tilde{\boldsymbol{\Pi}}}_{\boldsymbol{N}}=\left( \boldsymbol{I}_{\boldsymbol{N}}-\boldsymbol{A} \right)^{-\mathbf{1}}\boldsymbol{v}$,

which could be solved numerically.

One can also calculate the moments of the stationary distribution ${\tilde{\boldsymbol{\Pi}}}_{\boldsymbol{N}}$ (although ${\tilde{\boldsymbol{\Pi}}}_{\boldsymbol{N}}$ is not a probability distribution since it does not normalize to 1) by using Eq. S21 for the stationary distribution:

1. $M_{k}\left( {\tilde{\boldsymbol{\Pi}}}_{\boldsymbol{N}} \right)=\sum_{i=1}^{N} \tilde{\Pi}_{Ni}i^{k}=mN+\sum_{j=1}^{N} M_{k}\left( \boldsymbol{B}_{N}\left( \left( 1-m \right)\frac{j}{N} \right) \right)\tilde{\Pi}_{Nj},$

where $M_{k}\left( \boldsymbol{B}_{N}\left( \left( 1-m \right)\frac{j}{N} \right) \right)$ is the k-th moment of the binomial distribution with $N$ experiments and a choice probability of $\left( 1-m \right)\frac{j}{N}$.

Using Eq. S23 we find that the first three moments are:

1. $M_{1}\left( {\tilde{\boldsymbol{\Pi}}}_{\boldsymbol{N}} \right)=N$
2. $M_{2}\left( {\tilde{\boldsymbol{\Pi}}}_{\boldsymbol{N}} \right)\equiv N^{2}r_{P}=\frac{N^{2}}{N-\left( N-1 \right)\left( 1-m \right)^{2}}$
3. $M_{3}\left( {\tilde{\boldsymbol{\Pi}}}_{\boldsymbol{N}} \right)\equiv N^{3}s_{P}=N^{3}\frac{\frac{N+2\left( N-1 \right)\left( 1-m \right)^{2}}{N-\left( N-1 \right)\left( 1-m \right)^{2}}}{N^{2}-\left( N-1 \right)\left( N-2 \right)\left( 1-m \right)^{3}}$,

where we used the notation of $r_{P}, s_{P}$ as used by Gardner & West (2010) [8]. The same moments or very similar ones have been used also by many others (e.g., [4, 5]).

For the two-size patch distribution, the population measures of clonality level, general, and non-clonal relatedness depend on the parameters $\epsilon,N,m$ in a complicated way. For the full migration ($m=1$) case we show in the manuscript, these measures are simply: $x_{1c}=1-\epsilon, r=1-\epsilon+\frac{\epsilon}{N}, \rho=\frac{1}{N}$. However, for smaller migration levels, there is some level of clonality also in the larger patches. One can show that this amounts to $x_{1c}=1-\epsilon+\epsilon\tilde{\Pi}_{NN}, r=1-\epsilon+\epsilon r_{P}, \rho=\frac{r_{P}-\tilde{\Pi}_{NN}}{1-\tilde{\Pi}_{NN}}.$

### Using the stationary patch distribution in the Price equation

In the case of weak selection, the rate of change in the frequency $p_{1}$ is much slower than the rate by which the patch distribution reaches the stationary one (Eq. S21). We can therefore use this stationary distribution, to write Price’s equation (Eq. S1). For this purpose, a population average of a quantity $X$ is calculated in the following way:

1. $\left\langle X \right\rangle=\epsilon\sum_{j=0}^{N} P_{Nj}\left\langle X \right\rangle_{Nj}+\left( 1-\epsilon\right)\left( \left( 1-p_{1} \right)\left\langle X \right\rangle_{10}+p_{1}\left\langle X \right\rangle_{11} \right)$,

where $\left\langle X \right\rangle_{1j}$ and $\left\langle X \right\rangle_{Nj}$ are averages of the quantity $X$ within a patch of size $1$ or $N$ while $j$ of them are of the invader genotype. We use the same notations as in the general model (Eq. S1) for $g,W\left( g,G,N \right),W_{i}\left( G,N \right)$ (the relevant environmental parameter is now the inoculum size $N$) and obtain Price’s equation:

1. $\frac{\Delta p_{1}}{p_{1}}=\epsilon\sum_{j=1}^{N} \frac{j}{N}\tilde{\Pi}_{Nj}W_{1}\left( \frac{j}{N},N \right)+\left( 1-\epsilon\right)W_{1}\left( 1,1 \right)-1+O\left( p_{1} \right)$.

#### Calculating fitness in a given patch composition

We therefore need to calculate $W_{1}$ by examining the relation between fecundity and fitness in this infinite island scheme. The absolute fitness (equals to relative fitness since $\left\langle W \right\rangle=1$) of an invader in a patch with $j$ invaders out of $N$ is a sum of two terms:

1. $W_{1}\left( \frac{j}{N},N \right)=N\frac{\left( 1-m \right)\left( 1+f_{1}\left( \frac{j}{N} \right) \right)}{N\left( 1-m \right)\left( 1+f_{a} \right)+Nm\left( 1+f_{2}\left( 0 \right) \right)}+T\frac{m\left( 1+f_{1}\left( \frac{j}{N} \right) \right)}{T\left( 1+f_{2}\left( 0 \right) \right)}+O\left( p_{1} \right)$.

The first term is equal to the average number of its descendants in the same patch in the next generation, so it is $N$ multiplied by their fraction before sampling. The numerator is proportional to the number of its descendants that did not migrate. The denominator counts both the descendants from this patch that did not migrate (where $f_{a}\left( \frac{j}{N} \right)=\left( \frac{j}{N}f_{1}\left( \frac{j}{N} \right)+\frac{N-j}{N}f_{2}\left( \frac{j}{N} \right) \right)$ is the average fecundity within this patch) and immigrants to this patch from all other patches. In the limit where genotype $\#1$ is rare, we can neglect immigrants that come from clonal invader patches or mixed patches, since their contribution is of order $p_{1}$. The second term is the number of its descendants in all the other patches, so it’s the total population size after sampling, $T$, multiplied by their fraction in the overall population. Again, clonal invader patches and mixed patches are neglected. Keeping terms up to first order in $f_{i}\left( \frac{j}{N} \right)$, and substituting $f_{2}\left( 0 \right)=0$, we get:

1. $W_{1}\left( \frac{j}{N},N \right)=1+f_{1}\left( \frac{j}{N} \right)-\left( 1-m \right)^{2}f_{a}\left( \frac{j}{N} \right)+O\left( p_{1},f_{i}^{2} \right)$.

Note that for the case of limited migration the fitness of genotype #1 depends also on the fecundity of genotype #2 within the patch. This is different from the case of full migration ($m=1$), where the fecundity equals fitness and Price’s equation is independent of the fecundity of genotype #2 in mixed patches. For $j=N=1$ we get the fitness of an invader in a clonal patch:

1. $W_{1}\left( 1,1 \right)=1+f_{1}\left( 1 \right)m\left( 2-m \right)+O\left( p_{1},f_{i}^{2} \right)$.

Similar calculations for genotype $\#2$ validate that the mean fitness in the population $\left\langle W \right\rangle\approx\left\langle W_{2} \right\rangle$ is equal to 1. The relative change in the frequency of the invader is therefore:

1. $\frac{\Delta p_{1}}{p_{1}}=\epsilon\sum_{j=1}^{N} \frac{j}{N}\tilde{\Pi}_{Nj}\left( f_{1}\left( \frac{j}{N} \right)-\left( 1-m \right)^{2}f_{a} \right)+\left( 1-\epsilon\right)f_{1}\left( 1 \right)\left( 2m-m^{2} \right)$.

For a general non-linear fecundity function, Eq. S22 can be solved numerically to yield the stationary distribution ${\tilde{\boldsymbol{\Pi}}}_{\boldsymbol{N}}$ to be substituted in Eq. S32.

Alternatively, if the fecundity functions are expressed as power series as a function of the frequency $G$ of invaders in a social group:

1. $f_{1}\left( G \right)=\sum_{k=0}^{\infty} f_{1k}G^{k}; f_{a}\left( G \right) =\sum_{k=0}^{\infty} f_{ak}G^{k}$.

We find that Eq. S32 can be written as:

1. $\frac{\Delta p_{1}}{p_{1}}=\epsilon\sum_{k=0}^{\infty} \left( f_{1k}-\left( 1-m \right)^{2}f_{ak} \right)\frac{M_{k+1}\left( {\tilde{\boldsymbol{\Pi}}}_{\boldsymbol{N}} \right)}{N_{k+1}}+\left( 1-\epsilon\right)f_{1}\left( 1 \right)\left( 2m-m^{2} \right)$.

### Analytical derivation of the invasion conditions for mutual facultative cooperation and exploitive interactions

To understand the patterns of invasions between a cheater and two facultative cooperators, one needs to define the fecundity as a function of the invader frequency, $G$. This is done for three scenarios: invasion of a cheater into a facultative cooperator, invasion of a facultative cooperator into a cheater and invasion of one facultative cooperator into the other (since the facultative cooperators are symmetrical, there is no need to consider the opposite case).

Our assumptions in calculating the fecundity functions are that the public good molecule is produced by each facultative cooperator in a level proportional to its frequency in the population, so the total benefit of the public good produced by each facultative cooperator is proportional to the square of its frequency, while the cost of producing the public good is proportional to the frequency. $B,C$ are used as parameters for the benefit and cost of full (population wide) public good production. We subtract a constant value from the fecundity of both genotypes to set $f_{2}\left( 0 \right)=0$. The resulting fecundity functions are shown in Table S1. The fecundity functions are second order polynomials of the frequency, and we can therefore use the first three moments, defined in Eq. S24-S26, and Eq. S34 to find the relative change in frequency in the different scenarios (Table S2, and Fig. S1). Finally, we can use the three expressions for the change in frequency to derive the invasion condition for each scenario, given in Table S3.

We therefore find that facultative-cooperation allele diversity would be maintained while avoiding invasion by cheaters if:

1. $\frac{\epsilon\left( 1-\left( 1-m \right)^{2}\left( 2r_{P}-s_{P} \right) \right)+\left( 1-\epsilon\right)m\left( 2-m \right)}{\left( \epsilon\left( 2r_{P}-s_{P} \right)+1-\epsilon\right)m\left( 2-m \right)}<\frac{B}{C}<\frac{1-r_{P}-2\left( 1-m \right)^{2}\left( r_{P}-s_{P} \right)}{2\left( r_{P}-s_{P} \right)m\left( 2-m \right)}$

For the simple case of full-migration this condition reduces to:

1. $\frac{N^{2}}{\epsilon\left( 2N-1 \right)+N^{2}\left( 1-\epsilon\right)}<\frac{B}{C}<\frac{N}{2}$

This condition cannot be met in non-clonal conditions ($\epsilon=1$), but as $\epsilon\to0$ it is turned into:

1. $1+\epsilon\left( \frac{N-1}{N} \right)^{2}<\frac{B}{C}<\frac{N}{2}$

For limited migration the condition becomes more complicated.

### Infinite-island model with a distribution of patch sizes

In this section we generalize the previous analysis of two patch inoculum sizes to a distribution of patch sizes. We assume that $1-\epsilon$ of individuals singly inoculate one-founder patches, and the rest inoculate bigger patches ($2\leq N\leq N_{max}$) – with a probability $\epsilon a_{N}$ to inoculate a patch with $N$ founders ($\sum_{N=2}^{N_{max}} a_{N}=1$). The analysis of the stationary distributions of patch composition ${\tilde{\boldsymbol{\Pi}}}_{\boldsymbol{N}}$, for each $N$, follows the same analysis for the simpler case with one $N$ (Eq. S22, and Eqs. S23-S26 for the moments $M_{k}\left( {\tilde{\boldsymbol{\Pi}}}_{\boldsymbol{N}} \right)$). The calculation of $W_{1}\left( \frac{j}{N},N \right)$ for any $1\leq N\leq N_{max}$ is also identical to the calculation for the simpler model (Eq. S29). The calculation of a population average of a quantity $X$ changes to:

1. $\left\langle X \right\rangle=\epsilon\sum_{N=2}^{N_{max}} a_{N}\sum_{j=0}^{N} P_{Nj}\left\langle X \right\rangle_{Nj}+\left( 1-\epsilon\right)\left( \left( 1-p_{1} \right)\left\langle X \right\rangle_{10}+p_{1}\left\langle X \right\rangle_{11} \right)$,

where $\left\langle X \right\rangle_{Nj}$ are averages of the quantity $X$ within a patch of $N$ founders while $j$ of them are of the invader genotype. Therefore, the first term in the Eq. for the relative change in invader frequency is just a weighted average of the terms for each $N$:

1. $\frac{\Delta p_{1}}{p_{1}}=\epsilon\sum_{N=2}^{N_{max}} a_{N}\sum_{j=1}^{N} \frac{j}{N}\tilde{\Pi}_{Nj}\left( f_{1}\left( \frac{j}{N} \right)-\left( 1-m \right)^{2}f_{a} \right)+\left( 1-\epsilon\right)f_{1}\left( 1 \right)\left( 2m-m^{2} \right)$.

In the simpler two patch-size case, $N$ should be high enough for the invasion of a new facultative-cooperator allele to succeed. We therefore predict that in the current model, facultative-cooperator allele invasion would succeed if the probability to get large $N$ patches is high enough. Fig. S2 shows the relative change in the invader frequency for the case where the distribution of patch sizes $a_{N}$ is geometric with a cutoff by $N_{max}$ (for numerical implementation): $a_{N}=As\left( 1-s \right)^{N-2}$. $A$ is a normalization factor (depending on $s$ and $N_{max}$). This distribution is decreasing with $N$, and the rate of decrease becomes smaller as $s$ becomes smaller. When $N_{max}$ is small ($N_{max}=5$) the effect of the smaller $N$’s overtakes the effect of the larger $N$’s and a rare facultative cheater cannot invade independently of the geometric distribution parameter $s$. However, for $N_{max}\geq10$ the dependency on $s$ and $\epsilon$ converges and invasion of a rare facultative-cooperator allele succeeds when the speed of decrease $s$ is low enough. As in the simpler model, invasion of cheaters is prevented when there is either high clonality ($\epsilon$ is small) or high non-clonal relatedness ($s$ is large), and there is a region where the two occur simultaneously.

### The impact of clonality on the evolution of resistance to a facultative-harming greenbeard

As another example for a case where clonality affects the maintenance of kin-discriminative behaviors, we consider the case of facultative-harming greenbeards introduced by Gardner & West [8]. This type of greenbeard organism will engage in a harming behavior when it encounters a non-beard organism, but would not try to harm another greenbeard. At each harmful encounter, the greenbeard pays a cost $A$ but inflicts a larger harm $D$ on the non-beard. The interaction of a facultative-harming greenbeard with a non-beard is kin-discriminative, as both genotypes are not affected by the beard in clonal populations.

Here we consider the effect of a resistant organism on the greenbeard dynamics. A resistant organism is also attacked by a greenbeard organism but is not affected by this attack, because of a constitutive resistance, which incurs a cost $R$. The resistant strain's fecundity in a clonal group is therefore lower than that of the other two genotypes by the cost of resistance, $R$. Based on our analysis, we therefore predict that clonality would not affect the kin-discriminative interactions between the greenbeard and the non-beard, but would reduce the stability of the resistant strain.

We use the infinite island model described in the above section to analyze the ability of a rare genotype to invade a (single) resident genotype. The tables below follow the analysis done in the facultative cooperation case, and summarize the fecundity functions (Table S4), relative change in frequency (Table S5) and invasion conditions (Table S6) for four invasion scenarios. Two invasion scenarios have obvious results – the non-beard always invades the resistant and is never invaded by it – and are therefore not shown here.

As expected, the invasion of the greenbeard into the non-beard strain exactly matches the expression presented by West & Gardner in their supplementary material [8]. In contrast, as $\epsilon$ becomes smaller (a larger non-clonal population), the range of parameters for invasion of the greenbeard into the resistant strain is extended and the range of parameters for invasion of the resistant strain into the greenbeard is reduced.

If we assume an initial population of non-beard organisms and then consider their invasion by a greenbeard and, if succeeds, the invasion of a resistance strains, then we could end up in one of three states. 1) Dominance of non-beards (in conditions where the greenbeard cannot invade), 2) Dominance of greenbeards (in conditions where the greenbeard invades the non-beard but the resistant cannot invade the greenbeard), or 3) A rock-paper-scissor dynamics between the three genotypes (in conditions where the greenbeard invades the non-beard and the resistant invades the greenbeard). In Fig. S5 we illustrate the dependence of the three states on the migration level and clonality for specific values of the interaction parameters. We find that the distinction between a dominant non-beard state and other states is independent of clonality (as it is determined by the kin-discriminative interaction between the non-beard and the greenbeard). In contrast, increase in clonality increases the range of migration parameters, over which the greenbeard is dominant. This illustrates the importance of clonality for stabilization of the greenbeard state.

## Error estimation for experimental data analysis

Using the predint command in MATLAB Curve Fitting Toolbox R2015b (The MathWorks, Inc., Natick, Massachusetts) we calculated the upper and lower 68% prediction bounds (one sigma) of the fitness functions. Fig. S4A,B present these prediction bounds plotted on top of Fig. 3C,D of the main text, respectively. We then assumed errors were symmetric and independent and propagated them to obtain the errors for the invasion fitness, which is dictated by Eq. 5 of the main text. Fig. S4C,D present these propagated prediction bounds plotted on top of Fig. 3E,F of the main text, respectively.

## Accumulation of kin-discrimination loci

The main focus of our work is the maintenance of kin-discrimination diversity (and especially facultative cooperation diversity) in a single locus – i.e., how bacteria preserve functional kin-discriminating allelic diversity in a gene, where each bacterium carries a single copy of this gene. Another way by which kin-discrimination evolves is through the accumulation of multiple different kin-discriminative loci. Each of these loci can be absent (“null” allele) or present in one of multiple alleles in any given bacterium. Such accumulation of multiple kin-discriminative loci can lead to a combinatorial and highly refined level of kin-discrimination [13], as was observed in *E. coli* colicin systems [14], *B. subtilis* toxins [15, 16] and *B. subtilis* Rap-Phr quorum-sensing loci [17, 18].

The question of functional allelic diversity when considering each locus by itself is already dealt with in the manuscript. Here we ask what will be the effect of adding a novel locus to a strain, i.e., compare a strain with a functional allele to one with a “null” allele of the specific system. In contrast to competition between alleles, the cost of production of the kin-discrimination system lies only on the strain with the active allele. Therefore the question of whether clonal groups of the “null” and active strains have equal fitness (i.e., whether the interaction is kin-discriminative) depends on the extent of this cost. From a formal perspective, this problem is identical to the problem of invasion of a greenbeard allele into a non-beard population briefly discussed above and lengthily in ref. [8]. In the case of a toxin-antidote type of a kin-discrimination system, one can assume for simplicity that the cost of the system is negligible (which is probably not always true). In such a case, we expect that in well-mixed populations, the relative fitness of the two strain will be zero when the active locus is at low frequency (as the level of toxin is too low to hurt the “null” strain) and that the relative fitness of the active strain will become positive as its frequency increases. One can show that this will lead to invasion of the “active” strain under all population structures (as in Box Fig. A of the main text). If the system is costly, then this interaction is not kin-discriminative and an intermediate value of general relatedness will be needed for invasion of the system to occur [8].

In a previous experimental work, we have analyzed the invasion of a *B. subtilis* strain carrying an additional locus of the Rap-Phr system into a strain not carrying it [17] (Fig. S7A). The interaction in this case is of a facultative cooperator (active strain) against a constitutive cooperator (“null” strain) through the production of the public-good surfactant, Surfactin, during swarming motility, similar to what discussed in the main text. In Fig. S7 we show an analysis of this system done in a similar fashion to what was done in Fig. 3 of the main text. We calculated the fitness of each strain at different frequencies (Fig. S7B) and introduced it into a model of a population structure with varying levels of clonality and non-clonal relatedness to calculate the invasion probability (Fig. S7C). As can be seen, the interaction between the strains is kin-discriminative (the two clonal populations have equal swarm yields) implying a low cost of maintaining the quorum-sensing system). In addition, the fitness of the “active” strain with the additional quorum-sensing system is always (at any given frequency) higher than the clonal fitness of the “null” strain (Fig. S7B). This leads to selection for the “active” strain at any population structure (Fig. S7C).

## Supplementary text references

1. Price GR. Extension of covariance selection mathematics. Annals of human genetics. 1972;35(4):485-90.

2. Gardner A, West SA, Wild G. The genetical theory of kin selection. Journal of evolutionary biology. 2011;24(5):1020-43.

3. Smith j, Van Dyken JD, Zee PC. A Generalization of Hamilton’s Rule for the Evolution of Microbial Cooperation. Science. 2010;328(5986):1700-3.

4. Ohtsuki H. Evolutionary games in Wright's island model: kin selection meets evolutionary game theory. Evolution. 2010;64(12):3344-53.

5. Rousset F, Roze D. Constraints on the origin and maintenance of genetic kin recognition. Evolution. 2007;61(10):2320-30.

6. Roze D, Rousset F. Inbreeding depression and the evolution of dispersal rates: a multilocus model. The American Naturalist. 2005;166(6):708-21.

7. Lehmann L, Rousset F, Roze D, Keller L. Strong reciprocity or strong ferocity? A population genetic view of the evolution of altruistic punishment. The American Naturalist. 2007;170(1):21-36.

8. Gardner A, West SA. GREENBEARDS. Evolution. 2010;64(1):25-38.

9. Ackermann M, Stecher B, Freed NE, Songhet P, Hardt W-D, Doebeli M. Self-destructive cooperation mediated by phenotypic noise. Nature. 2008;454(7207):987-90.

10. Wild G, Gardner A, West SA. Adaptation and the evolution of parasite virulence in a connected world. Nature. 2009;459:983.

11. Roze D, Rousset F. Multilocus models in the infinite island model of population structure. Theoretical population biology. 2008;73(4):529-42.

12. Biernaskie J, Gardner A, West S. Multicoloured greenbeards, bacteriocin diversity and the rock‐paper‐scissors game. Journal of evolutionary biology. 2013;26(10):2081-94.

13. Strassmann JE, Gilbert OM, Queller DC. Kin discrimination and cooperation in microbes. Annual Review of Microbiology. 2011;65:349-67.

14. Gordon DM, O'Brien CL. Bacteriocin diversity and the frequency of multiple bacteriocin production in Escherichia coli. Microbiology. 2006;152(11):3239-44.

15. Stefanic P, Kraigher B, Lyons NA, Kolter R, Mandic-Mulec I. Kin discrimination between sympatric Bacillus subtilis isolates. Proceedings of the National Academy of Sciences. 2015:201512671.

16. Lyons NA, Kraigher B, Stefanic P, Mandic-Mulec I, Kolter R. A combinatorial kin discrimination system in Bacillus subtilis. Current Biology. 2016;26(6):733-42.

17. Pollak S, Bendori SO, Even-Tov E, Lipsman V, Bareia T, Ben-Zion I, et al. Facultative cheating supports the co-existence of multiple quorum-sensing pherotypes. Proceedings of the National Academy of Sciences. 2016;113(8):2152-7.

18. Even-Tov E, Omer Bendori S, Pollak S, Eldar A. Transient Duplication-Dependent Divergence and Horizontal Transfer Underlie the Evolutionary Dynamics of Bacterial Cell–Cell Signaling. PLoS biology. 2016;14(12):e2000330.

19. Even-Tov E, Bendori SO, Valastyan J, Ke X, Pollak S, Bareia T, et al. Social evolution selects for redundancy in bacterial quorum sensing. PLOS Biology. 2016;14(2):e1002386.

# Supplementary tables

**Table S1**: Fecundity functions for the three invasion scenarios, as a function of invader frequency in a well-mixed patch.

| **Invasion scenario** | **Fecundity functions** |
| --- | --- |
| Cheater into Facultative Cooperator | $f_{1}=B\left( 1-G \right)^{2}-\left( B-C \right)=-B\left( 2G-G^{2} \right)+C$  $f_{2}=B\left( 1-G \right)^{2}-C\left( 1-G \right)-\left( B-C \right)=-B\left( 2G-G^{2} \right)+CG$  $f_{a}=\left( C-B \right)\left( 2G-G^{2} \right)$ |
| Facultative Cooperator  into Cheater | $f_{1}=BG^{2}-CG$  $f_{2}=BG^{2}$  $f_{a}=\left( B-C \right)G^{2}$ |
| One Facultative Cooperator into another | $f_{1}=B\left( \left( 1-G \right)^{2}+G^{2} \right)-CG-\left( B-C \right)=-2B\left( G-G^{2} \right)+C(1-G)$  $f_{2}=B\left( \left( 1-G \right)^{2}+G^{2} \right)-C\left( 1-G \right)-\left( B-C \right)=-2B\left( G-G^{2} \right)+CG$  $f_{a}=\left( 2C-2B \right)\left( G-G^{2} \right)$ |

**Table S2**: The relative change in the frequency of the invader for the three invasion scenarios, based on Eq. S34.

| **Invasion scenario** | **Relative change in frequency,** $\frac{\boldsymbol{\Delta}\boldsymbol{p}_{\boldsymbol{1}}}{\boldsymbol{p}_{\boldsymbol{1}}}$ |
| --- | --- |
| Cheater into Facultative Cooperator | $\epsilon\left( C-\left( B-\left( 1-m \right)^{2}\left( B-C \right) \right)\left( 2r_{P}-s_{P} \right) \right)-\left( 1-\epsilon\right)m\left( 2-m \right)\left( B-C \right)$ |
| Facultative Cooperator  into Cheater | $\epsilon\left( Bs_{P}-Cr_{P}-\left( 1-m \right)^{2}\left( B-C \right)s_{P} \right)+\left( 1-\epsilon\right)m\left( 2-m \right)\left( B-C \right)$ |
| One Facultative Cooperator into another | $\epsilon\left( C\left( 1-r_{P} \right)-\left( 2B-\left( 1-m \right)^{2}\left( 2B-2C \right) \right)\left( r_{P}-s_{P} \right) \right)$ |

**Table S3**: Invasion conditions for the three scenarios.

| **Invasion scenario** | **Invasion condition** |
| --- | --- |
| Cheater into Facultative Cooperator | $\frac{B}{C}<\frac{\epsilon\left( 1-\left( 1-m \right)^{2}\left( 2r_{P}-s_{P} \right) \right)+\left( 1-\epsilon\right)m\left( 2-m \right)}{\left( \epsilon\left( 2r_{P}-s_{P} \right)+1-\epsilon\right)m\left( 2-m \right)}$ |
| Facultative Cooperator  into Cheater | $\frac{B}{C}>\frac{\epsilon\left( r_{P}-\left( 1-m \right)^{2}s_{P} \right)+\left( 1-\epsilon\right)m\left( 2-m \right)}{\left( \epsilon s_{P}+1-\epsilon\right)m\left( 2-m \right)}$ |
| One Facultative Cooperator into another | $\frac{B}{C}<\frac{1-r_{P}-2\left( 1-m \right)^{2}\left( r_{P}-s_{P} \right)}{2\left( r_{P}-s_{P} \right)m\left( 2-m \right)}$ |

**Table S4:** Fecundity functions for the four invasion scenarios, as a function of the invader frequency in a well-mixed patch.

| **Invasion scenario** | **Fecundity functions** |
| --- | --- |
| Greenbeard into Non-beard | $f_{1}=-A\left( 1-G \right)$  $f_{2}=-DG$  $f_{a}=-\left( A+D \right)\left( G-G^{2} \right)$ |
| Non-beard into Greenbeard | $f_{1}=-D\left( 1-G \right)$  $f_{2}=-AG$  $f_{a}=-\left( A+D \right)\left( G-G^{2} \right)$ |
| Resistant into Greenbeard | $f_{1}=-R$  $f_{2}=-AG$  $f_{a}=-A\left( G-G^{2} \right)-RG$ |
| Greenbeard into Resistant | $f_{1}=-A\left( 1-G \right)+R$  $f_{2}=0$  $f_{a}=-A\left( G-G^{2} \right)+RG$ |

**Table S5**: The relative change in the frequency of the invader for the four invasion scenarios, based on Eq. S34.

| **Invasion scenario** | **Relative change in frequency,** $\frac{\boldsymbol{\Delta}\boldsymbol{p}_{\boldsymbol{1}}}{\boldsymbol{p}_{\boldsymbol{1}}}$ |
| --- | --- |
| Greenbeard into Non-beard | $\epsilon\left( -A\left( 1-r_{P} \right)+\left( A+D \right)\left( 1-m \right)^{2}\left( r_{P}-s_{P} \right) \right)$ |
| Non-beard into Greenbeard | $\epsilon\left( -D\left( 1-r_{P} \right)+\left( A+D \right)\left( 1-m \right)^{2}\left( r_{P}-s_{P} \right) \right)$ |
| Resistant into Greenbeard | $\epsilon\left( -R\left( 1-\left( 1-m \right)^{2}r_{P} \right)+A\left( 1-m \right)^{2}\left( r_{P}-s_{P} \right) \right)-R\left( 1-\epsilon\right)m\left( 2-m \right)$ |
| Greenbeard into Resistant | $\epsilon\left( -A\left( 1-r_{P} \right)+R\left( 1-\left( 1-m \right)^{2}r_{P} \right)+A\left( 1-m \right)^{2}\left( r_{P}-s_{P} \right) \right)+R\left( 1-\epsilon\right)m\left( 2-m \right)$ |

**Table S6**: Invasion conditions for the four scenarios.

| **Invasion scenario** | **Invasion condition** |
| --- | --- |
| Greenbeard into Non-beard | $\frac{D}{A}>\frac{1-r_{P}}{\left( 1-m \right)^{2}\left( r_{P}-s_{P} \right)}-1$ |
| Non-beard into Greenbeard | $\frac{A}{D}>\frac{1-r_{P}}{\left( 1-m \right)^{2}\left( r_{P}-s_{P} \right)}-1 (condition never satisfied)$ |
| Resistant into Greenbeard | $\frac{R}{A}<\frac{\epsilon\left( 1-m \right)^{2}\left( r_{P}-s_{P} \right)}{\epsilon\left( 1-\left( 1-m \right)^{2}r_{P} \right)+\left( 1-\epsilon\right)m\left( 2-m \right)}$ |
| Greenbeard into Resistant | $\frac{R}{A}>\frac{\epsilon\left( \left( 1-r_{P} \right)-\left( 1-m \right)^{2}\left( r_{P}-s_{P} \right) \right)}{\epsilon\left( 1-\left( 1-m \right)^{2}r_{P} \right)+\left( 1-\epsilon\right)m\left( 2-m \right)}$ |

# Supplementary figures


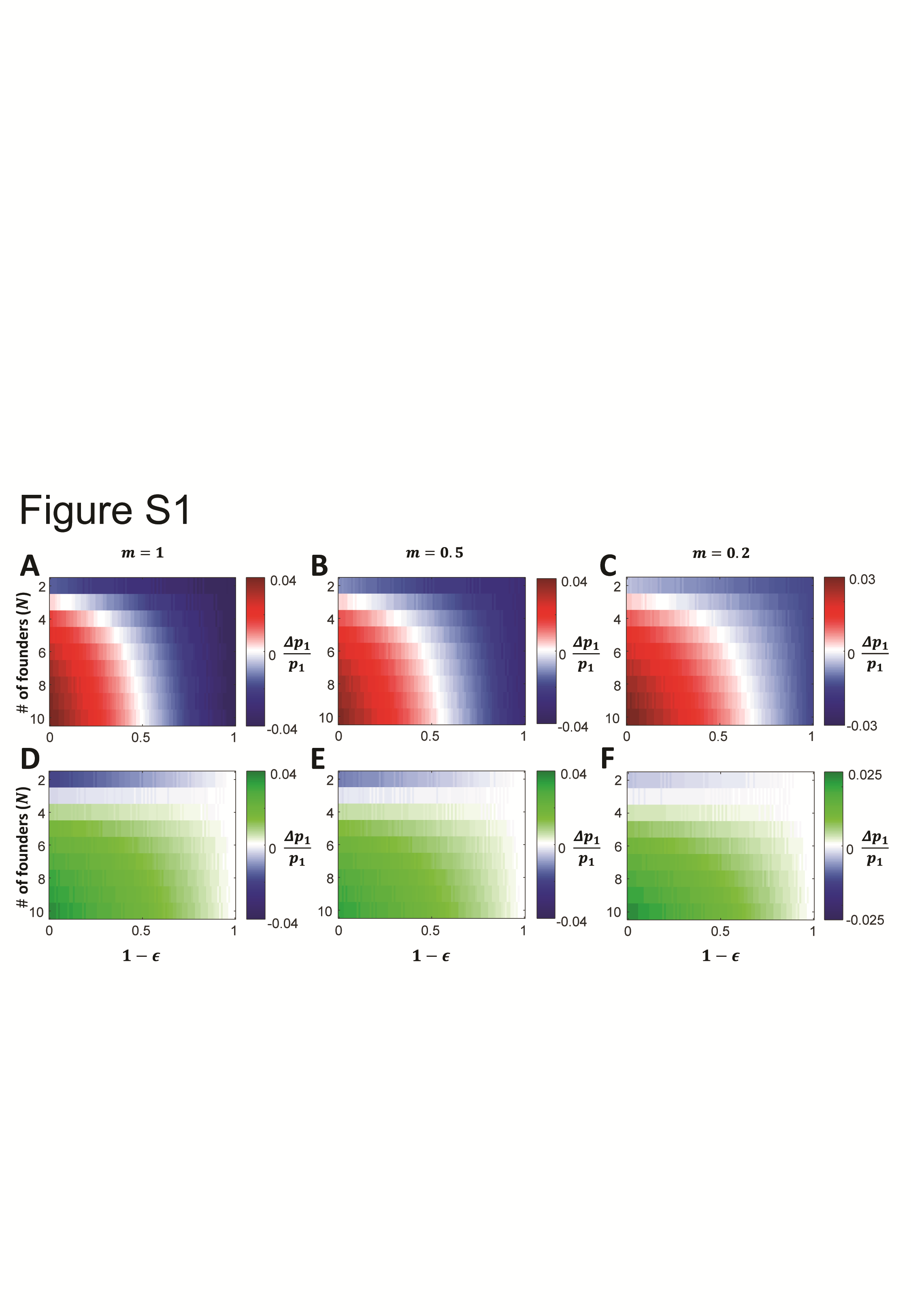


**Figure S1:** **Infinite island model with both clonal and non-clonal bottlenecks, with varying migration strength. (A-F)** Similar to Fig. 2D-E for the two interactions presented in Fig. 2A,B; Cheater invading a population of facultative cooperators **(A-C)**, and one facultative cooperator invading a population of another **(D-F)**. The only change is that migration strength, $m$, is varied; $m=1$ for (A,D), $m=0.5$ for (B,E), and $m=0.2$ for (C,F).


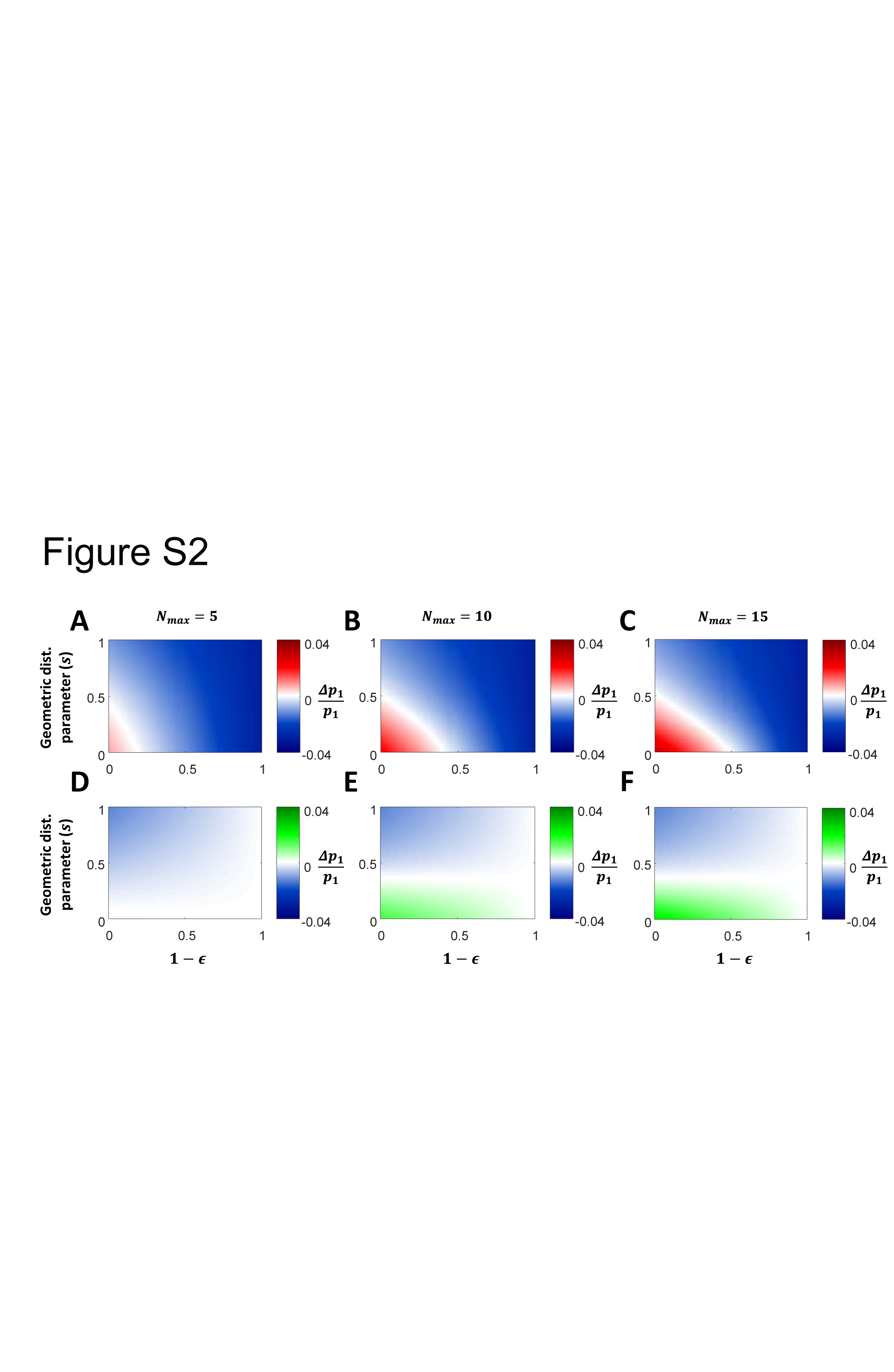


**Figure S2: Infinite island model with a monotonously decreasing patch-size distribution. (A-F)** Similar to Fig. 2D-E. Each graph shows the relative change in the frequency of the invader as a function of the clonality level $\epsilon$ and the geometric-distribution parameter $s$, for the two interactions presented in Fig. 2A,B; Cheater invading a population of facultative cooperators **(A-C)**, and one facultative cooperator invading a population of another **(D-F)**. The difference from Fig. 2D-E is that non-clonal patch size distribution $a_{N}$ is defined over the range $2\leq N\leq N_{max}$ via a geometric distribution, $a_{N}\propto s\left( 1-s \right)^{N-2}$ with a varying $N_{max}$ between panels; $N_{max}=5$ for (A,D), $N_{max}=10$ for (B,E), and $N_{max}=15$ for (C,F). The y-axis in each graph is the parameter $s$ of the geometric distribution – the lower $s$ is, the more heavy-tailed is the distribution.


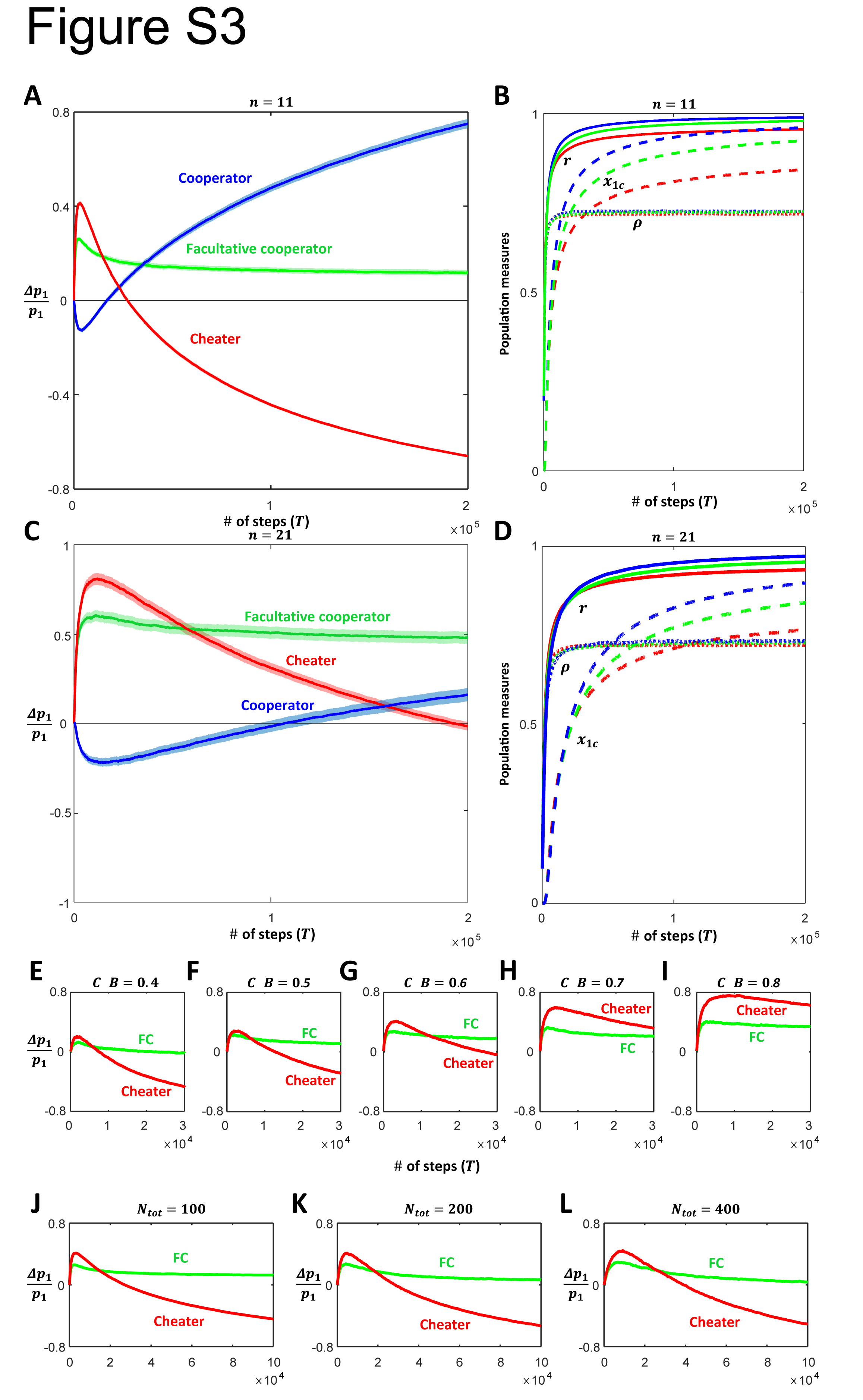


**Figure S3: Varying parameters for the colony growth simulations. (A-D)** Similar to Fig. 2G,H (main text), with a few changes. Data for the invasion of a facultative cooperator into a population of a cheater is also presented (in blue). Confidence regions ($\pm$ standard error) for the relative change in the invader’s frequency are presented by the shaded-color regions in (A,C). Population measures for all three interactions are presented in (B,D). The neighborhood size, $n$, is varied; $n=11$ (as in Fig. 2G,H) in (A,B), and $n=21$ in (C,D). The number of simulation repeats is $84,000$ in (A,B), and $16,000$ in (C,D). **(E-I)** Similar to Fig. 2G with three changes. The number of time steps is $T=30,000$, the number of simulation repeats is $10,000$, and the ratio between the cost and benefit parameters is varied from $\frac{C}{B}=0.4$ in (E) to $\frac{C}{B}=0.8$ in (I). **(J-L)** The initial colony size is changed such that the number of cells on the colony border is $N_{tot}=100$ in (J) (as in Fig. 2G), $N_{tot}=200$ in (K) and $N_{tot}=400$ in (L). The number of time steps is $T=100,000$ and the number of simulation repeats is 20$,000$.


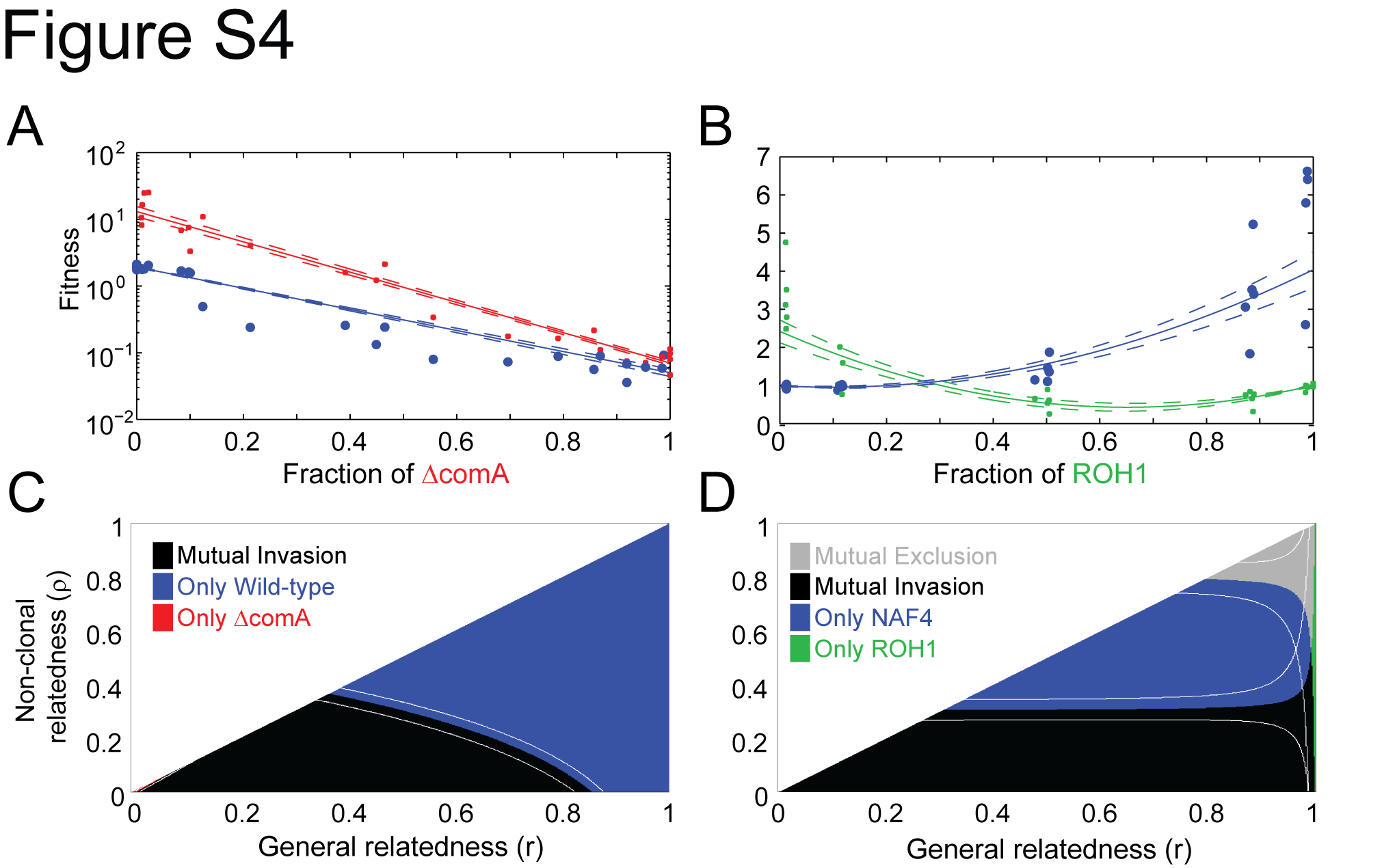


**Figure S4: Error estimation for figure 3 in the main text.** Presented are **(A-B)** Fig. 3C,D of the main text together with the corresponding 68% prediction bounds (one sigma, dashed lines) for the fitting functions, and **(C-D)** Fig. 3E,F of the main text together with their corresponding propagated prediction bounds (dashed lines represent sign change of the invasion fitness plus or minus its propagated error).


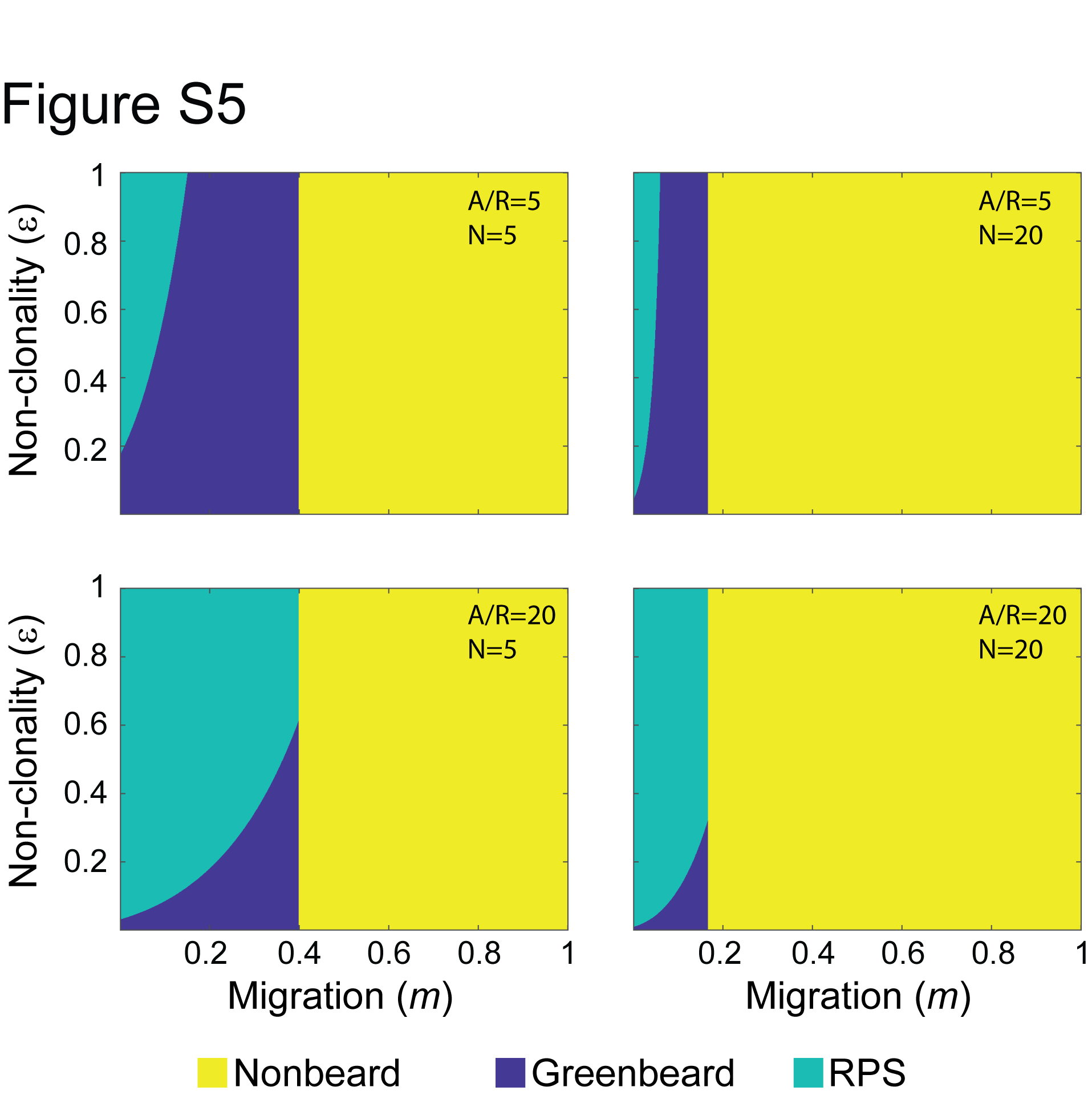


**Figure S5: Clonality stabilizes a facultative-harming greenbeard against invasion of a costly resistant strain.** Shown are phase plots of the expected dynamic behavior of a system of three genotypes: A non-beard, a facultative-harming greenbeard, which inflicts a harm $D$ with an encounter cost $A$ on a non-beard and a resistant strain which has a constitutive resistance cost $R$. Depending on invasion patterns, one can envision three major regions where non-beards dominate (yellow), where greenbeards dominate (blue), or where a rock-paper-scissor invasion dynamics occurs (resistant invades the greenbeard, which invades the non-beard, which invades the resistant, teal). Phase plots are shown as a function of non-clonality parameter $\epsilon$ and migration rate $m$, as described in Table S6. The different parameters reflect changes in the non-clonal patch size; $N=5$ (left), $N=20$ (right), and in the cost of resistance $\frac{A}{R}=5$ (top), $\frac{A}{R}=20$ (bottom). For high resistance cost, dominance of greenbeard is allowed even with no clonality ($\epsilon=1$). At low resistance cost, greenbeard dominance is achieved only at partial clonality. In all cases we assume that $\frac{D}{A}=10$.

**Figure S6: Colony growth simulations for positive frequency-dependent interactions. (A)** Frequency dependence of the fecundity of a microbe (of either genotype $\#1$, magenta, or genotype $\#2$, blue) in a well-mixed interaction group with a fraction $G$ of invaders (genotype $\#1$). The shown interaction has a positive frequency-dependent structure (selection for majority). It can arise from the presence of a different bacteriocin-immunity pair in each genotype **(B)** The relative change in invader frequency for varying initial number of invaders ($N_{i}=3,5,7$), with confidence regions ($\pm$ standard error) presented with shaded-color. The neighborhood size is $n=11$, and the number of simulation repeats is of the order of ${10}^{5}$. Selection is positive if $N_{i}=7$, i.e., invaders have high relatedness from the initial stages.


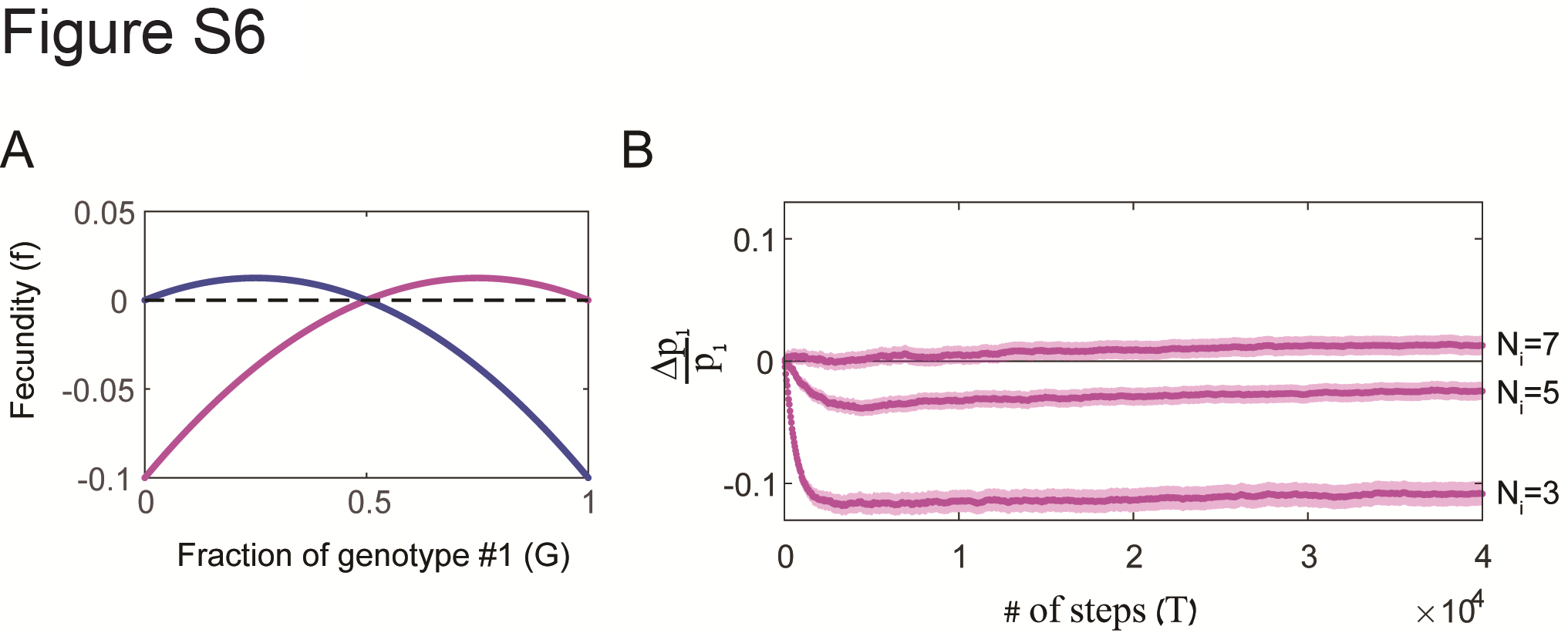

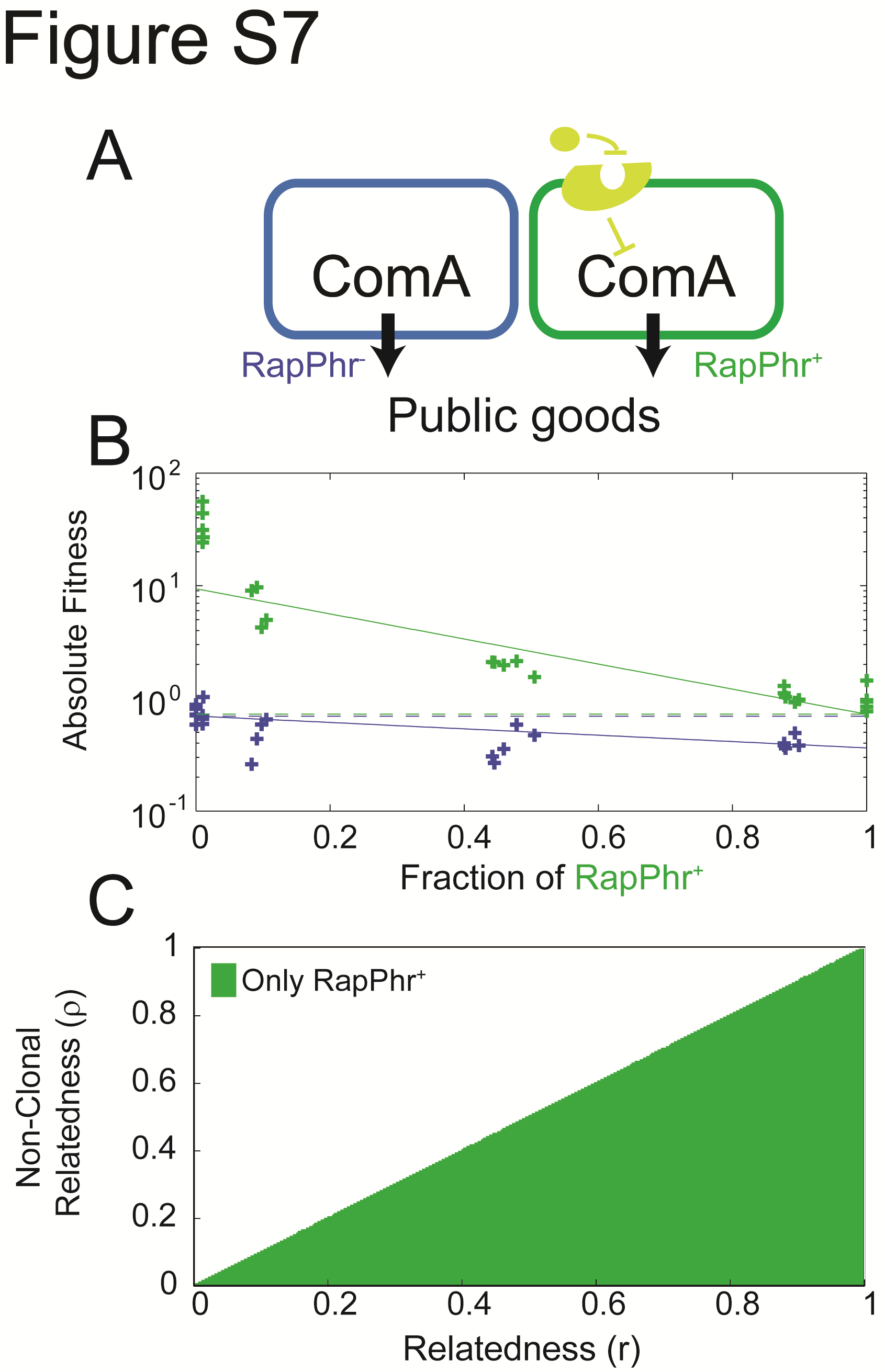


**Figure S7: Selection for an additional Rap-Phr system in *B. subtilis* is independent of population structure.** (**A**) A schematic illustration of two interacting strains, one including an additional Rap-Phr locus (Rap-Phr^+^) compared to the other (Rap-Phr^-^). Rap negatively regulates production of public goods through inhibition of ComA. Phr derepresses public good production by preventing Rap activity. (**B**) Fitness functions for the interaction between a Rap-Phr^+^ (green) and a Rap-Phr^-^ (blue) *B. subtilis* strains during swarming. Fitness was calculated as in Fig. 3 of the manuscript, based on relative frequency and yield results measured in ref. [19]. (**C**) Rap-Phr^+^ will always dominate the Rap-Phr^-^ strain in a structured population where non-clonal and clonal relatedness can be determined individually. See Methods section in the main text for a description of the structured population.
