# Supplementary material for: Clonality and non-linearity drive facultative-cooperation allele diversity
Source: ISME J. 2018 Nov 21;13(3):824–35. doi: 10.1038/s41396-018-0310-y (PMC6461992; doi:10.1038/s41396-018-0310-y)
Supplement: Supplementary file 2 — Table S1 [file 41396_2018_310_MOESM2_ESM.docx]

**Table S1**: Fecundity functions for the three invasion scenarios, as a function of invader frequency in a well-mixed patch.

| **Invasion scenario** | **Fecundity functions** |
| --- | --- |
| Cheater into Facultative Cooperator | $f_{1}=B\left( 1-G \right)^{2}-\left( B-C \right)=-B\left( 2G-G^{2} \right)+C$  $f_{2}=B\left( 1-G \right)^{2}-C\left( 1-G \right)-\left( B-C \right)=-B\left( 2G-G^{2} \right)+CG$  $f_{a}=\left( C-B \right)\left( 2G-G^{2} \right)$ |
| Facultative Cooperator  into Cheater | $f_{1}=BG^{2}-CG$  $f_{2}=BG^{2}$  $f_{a}=\left( B-C \right)G^{2}$ |
| One Facultative Cooperator into another | $f_{1}=B\left( \left( 1-G \right)^{2}+G^{2} \right)-CG-\left( B-C \right)=-2B\left( G-G^{2} \right)+C(1-G)$  $f_{2}=B\left( \left( 1-G \right)^{2}+G^{2} \right)-C\left( 1-G \right)-\left( B-C \right)=-2B\left( G-G^{2} \right)+CG$  $f_{a}=\left( 2C-2B \right)\left( G-G^{2} \right)$ |
