# Supplementary material for: Clonality and non-linearity drive facultative-cooperation allele diversity
Source: ISME J. 2018 Nov 21;13(3):824–35. doi: 10.1038/s41396-018-0310-y (PMC6461992; doi:10.1038/s41396-018-0310-y)
Supplement: Supplementary file 3 — Table S2 [file 41396_2018_310_MOESM3_ESM.docx]

**Table S2**: The relative change in the frequency of the invader for the three invasion scenarios, based on Eq. S34.

| **Invasion scenario** | **Relative change in frequency,** $\frac{\boldsymbol{\Delta}\boldsymbol{p}_{\boldsymbol{1}}}{\boldsymbol{p}_{\boldsymbol{1}}}$ |
| --- | --- |
| Cheater into Facultative Cooperator | $\epsilon\left( C-\left( B-\left( 1-m \right)^{2}\left( B-C \right) \right)\left( 2r_{P}-s_{P} \right) \right)-\left( 1-\epsilon\right)m\left( 2-m \right)\left( B-C \right)$ |
| Facultative Cooperator  into Cheater | $\epsilon\left( Bs_{P}-Cr_{P}-\left( 1-m \right)^{2}\left( B-C \right)s_{P} \right)+\left( 1-\epsilon\right)m\left( 2-m \right)\left( B-C \right)$ |
| One Facultative Cooperator into another | $\epsilon\left( C\left( 1-r_{P} \right)-\left( 2B-\left( 1-m \right)^{2}\left( 2B-2C \right) \right)\left( r_{P}-s_{P} \right) \right)$ |
