# Supplementary material for: Clonality and non-linearity drive facultative-cooperation allele diversity
Source: ISME J. 2018 Nov 21;13(3):824–35. doi: 10.1038/s41396-018-0310-y (PMC6461992; doi:10.1038/s41396-018-0310-y)
Supplement: Supplementary file 4 — Table S3 [file 41396_2018_310_MOESM4_ESM.docx]

**Table S3**: Invasion conditions for the three scenarios.

| **Invasion scenario** | **Invasion condition** |
| --- | --- |
| Cheater into Facultative Cooperator | $\frac{B}{C}<\frac{\epsilon\left( 1-\left( 1-m \right)^{2}\left( 2r_{P}-s_{P} \right) \right)+\left( 1-\epsilon\right)m\left( 2-m \right)}{\left( \epsilon\left( 2r_{P}-s_{P} \right)+1-\epsilon\right)m\left( 2-m \right)}$ |
| Facultative Cooperator  into Cheater | $\frac{B}{C}>\frac{\epsilon\left( r_{P}-\left( 1-m \right)^{2}s_{P} \right)+\left( 1-\epsilon\right)m\left( 2-m \right)}{\left( \epsilon s_{P}+1-\epsilon\right)m\left( 2-m \right)}$ |
| One Facultative Cooperator into another | $\frac{B}{C}<\frac{1-r_{P}-2\left( 1-m \right)^{2}\left( r_{P}-s_{P} \right)}{2\left( r_{P}-s_{P} \right)m\left( 2-m \right)}$ |
