# Supplementary material for: Clonality and non-linearity drive facultative-cooperation allele diversity
Source: ISME J. 2018 Nov 21;13(3):824–35. doi: 10.1038/s41396-018-0310-y (PMC6461992; doi:10.1038/s41396-018-0310-y)
Supplement: Supplementary file 5 — Table S4 [file 41396_2018_310_MOESM5_ESM.docx]

**Table S4:** Fecundity functions for the four invasion scenarios, as a function of the invader frequency in a well-mixed patch.

| **Invasion scenario** | **Fecundity functions** |
| --- | --- |
| Greenbeard into Non-beard | $f_{1}=-A\left( 1-G \right)$  $f_{2}=-DG$  $f_{a}=-\left( A+D \right)\left( G-G^{2} \right)$ |
| Non-beard into Greenbeard | $f_{1}=-D\left( 1-G \right)$  $f_{2}=-AG$  $f_{a}=-\left( A+D \right)\left( G-G^{2} \right)$ |
| Resistant into Greenbeard | $f_{1}=-R$  $f_{2}=-AG$  $f_{a}=-A\left( G-G^{2} \right)-RG$ |
| Greenbeard into Resistant | $f_{1}=-A\left( 1-G \right)+R$  $f_{2}=0$  $f_{a}=-A\left( G-G^{2} \right)+RG$ |
