# Supplementary material for: Clonality and non-linearity drive facultative-cooperation allele diversity
Source: ISME J. 2018 Nov 21;13(3):824–35. doi: 10.1038/s41396-018-0310-y (PMC6461992; doi:10.1038/s41396-018-0310-y)
Supplement: Supplementary file 6 — Table S5 [file 41396_2018_310_MOESM6_ESM.docx]

**Table S5**: The relative change in the frequency of the invader for the four invasion scenarios, based on Eq. S34.

| **Invasion scenario** | **Relative change in frequency,** $\frac{\boldsymbol{\Delta}\boldsymbol{p}_{\boldsymbol{1}}}{\boldsymbol{p}_{\boldsymbol{1}}}$ |
| --- | --- |
| Greenbeard into Non-beard | $\epsilon\left( -A\left( 1-r_{P} \right)+\left( A+D \right)\left( 1-m \right)^{2}\left( r_{P}-s_{P} \right) \right)$ |
| Non-beard into Greenbeard | $\epsilon\left( -D\left( 1-r_{P} \right)+\left( A+D \right)\left( 1-m \right)^{2}\left( r_{P}-s_{P} \right) \right)$ |
| Resistant into Greenbeard | $\epsilon\left( -R\left( 1-\left( 1-m \right)^{2}r_{P} \right)+A\left( 1-m \right)^{2}\left( r_{P}-s_{P} \right) \right)-R\left( 1-\epsilon\right)m\left( 2-m \right)$ |
| Greenbeard into Resistant | $\epsilon\left( -A\left( 1-r_{P} \right)+R\left( 1-\left( 1-m \right)^{2}r_{P} \right)+A\left( 1-m \right)^{2}\left( r_{P}-s_{P} \right) \right)+R\left( 1-\epsilon\right)m\left( 2-m \right)$ |
