# Supplementary material for: Clonality and non-linearity drive facultative-cooperation allele diversity
Source: ISME J. 2018 Nov 21;13(3):824–35. doi: 10.1038/s41396-018-0310-y (PMC6461992; doi:10.1038/s41396-018-0310-y)
Supplement: Supplementary file 7 — Table S6 [file 41396_2018_310_MOESM7_ESM.docx]

**Table S6**: Invasion conditions for the four scenarios.

| **Invasion scenario** | **Invasion condition** |
| --- | --- |
| Greenbeard into Non-beard | $\frac{D}{A}>\frac{1-r_{P}}{\left( 1-m \right)^{2}\left( r_{P}-s_{P} \right)}-1$ |
| Non-beard into Greenbeard | $\frac{A}{D}>\frac{1-r_{P}}{\left( 1-m \right)^{2}\left( r_{P}-s_{P} \right)}-1 (condition never satisfied)$ |
| Resistant into Greenbeard | $\frac{R}{A}<\frac{\epsilon\left( 1-m \right)^{2}\left( r_{P}-s_{P} \right)}{\epsilon\left( 1-\left( 1-m \right)^{2}r_{P} \right)+\left( 1-\epsilon\right)m\left( 2-m \right)}$ |
| Greenbeard into Resistant | $\frac{R}{A}>\frac{\epsilon\left( \left( 1-r_{P} \right)-\left( 1-m \right)^{2}\left( r_{P}-s_{P} \right) \right)}{\epsilon\left( 1-\left( 1-m \right)^{2}r_{P} \right)+\left( 1-\epsilon\right)m\left( 2-m \right)}$ |
