# Supplementary figures and images for: Clonality and non-linearity drive facultative-cooperation allele diversity
Source: ISME J. 2018 Nov 21;13(3):824–35. doi: 10.1038/s41396-018-0310-y (PMC6461992; doi:10.1038/s41396-018-0310-y)

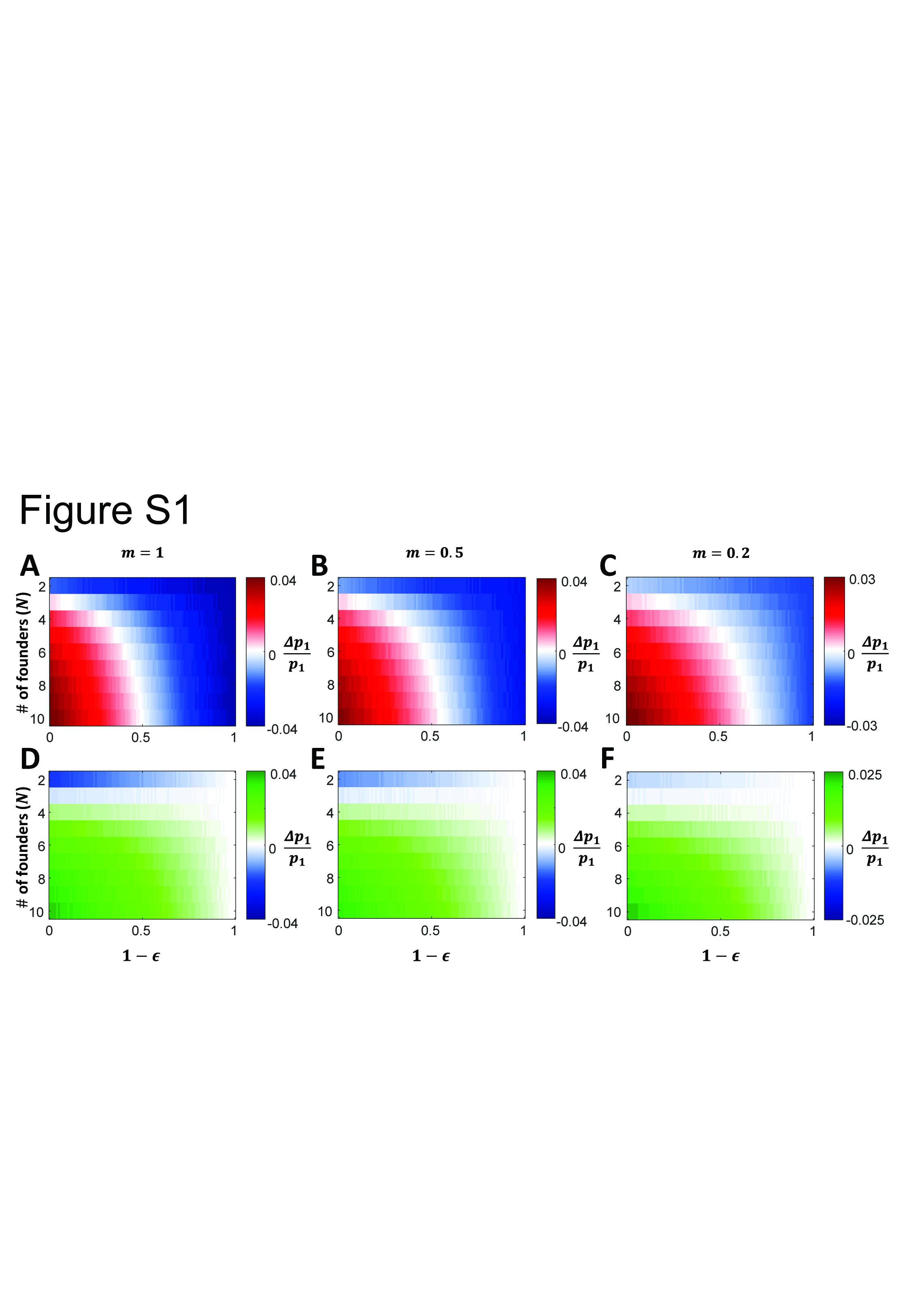

Supplement: Supplementary file 8 — Figure S1 [file 41396_2018_310_MOESM8_ESM.tif]

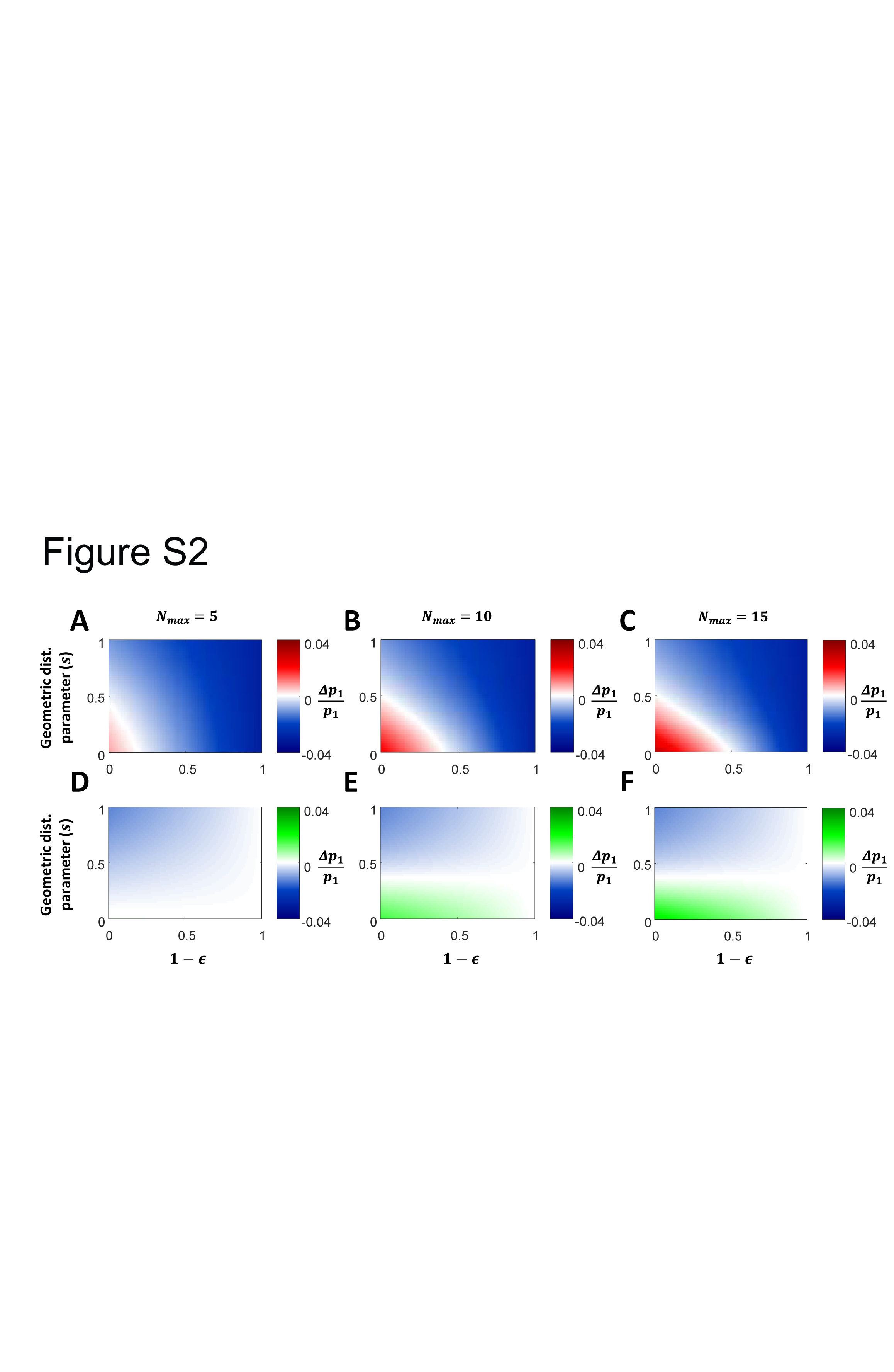

Supplement: Supplementary file 9 — Figure S2 [file 41396_2018_310_MOESM9_ESM.tif]

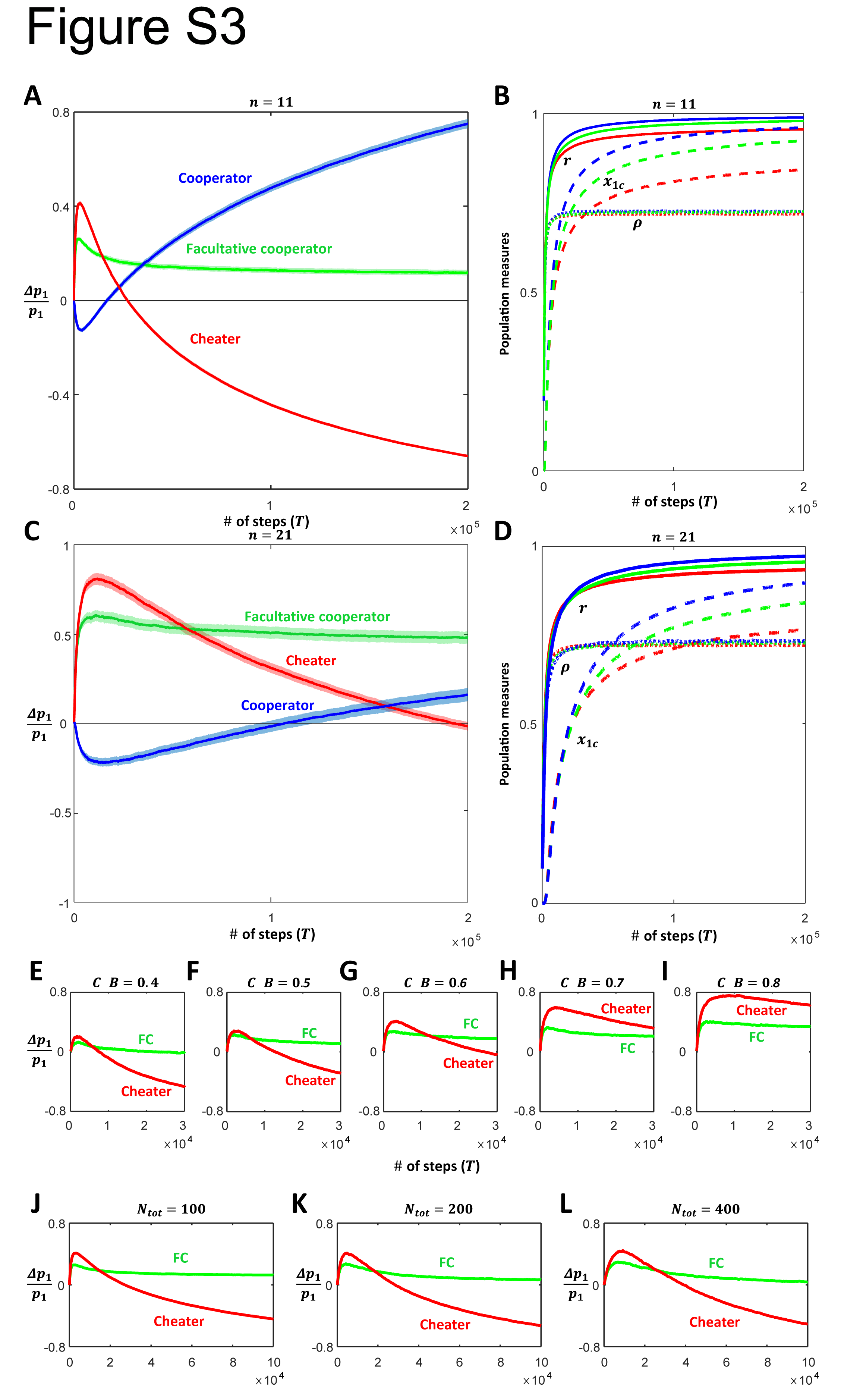

Supplement: Supplementary file 10 — Figure S3 [file 41396_2018_310_MOESM10_ESM.tif]

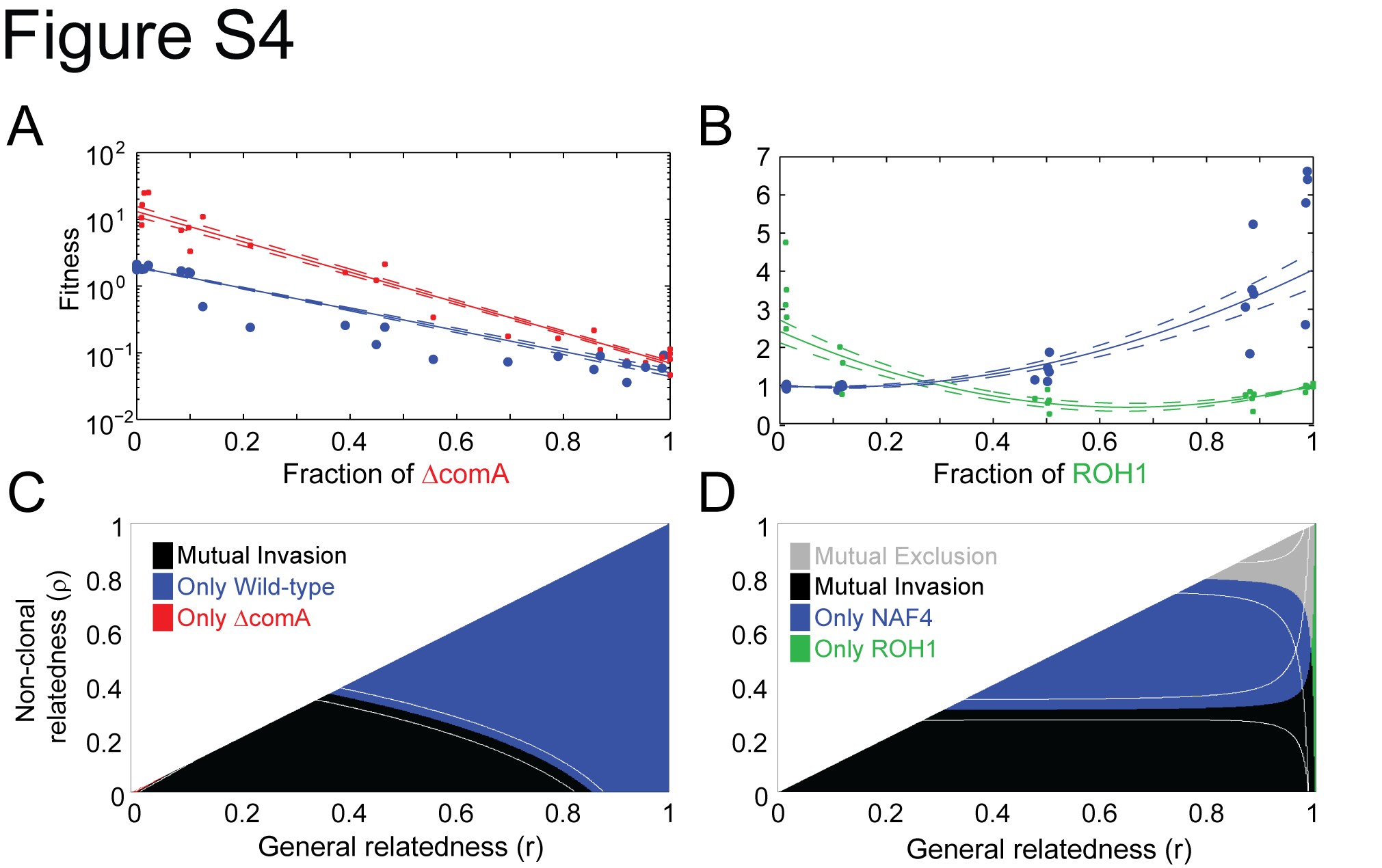

Supplement: Supplementary file 11 — Figure S4 [file 41396_2018_310_MOESM11_ESM.tif]

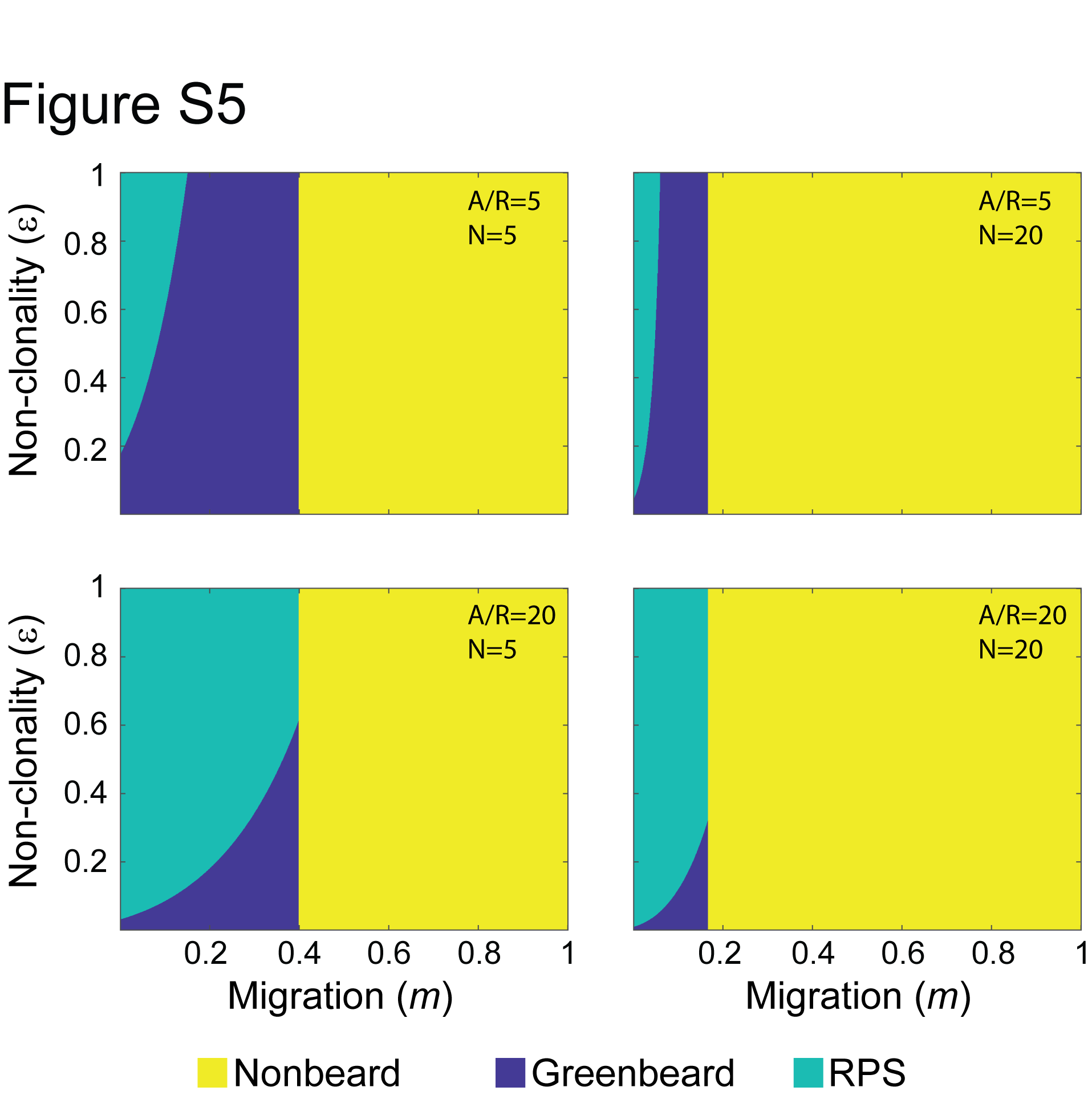

Supplement: Supplementary file 12 — Figure S5 [file 41396_2018_310_MOESM12_ESM.tif]

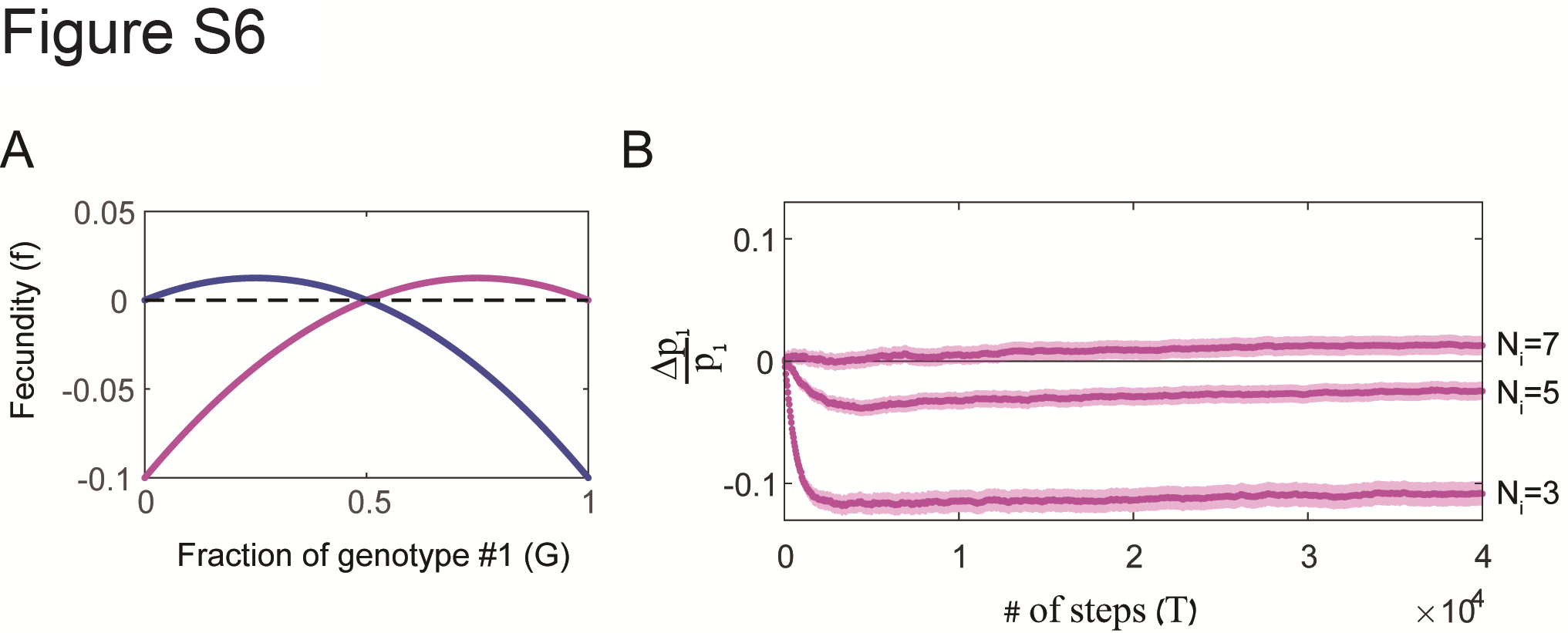

Supplement: Supplementary file 13 — Figure S6 [file 41396_2018_310_MOESM13_ESM.tif]

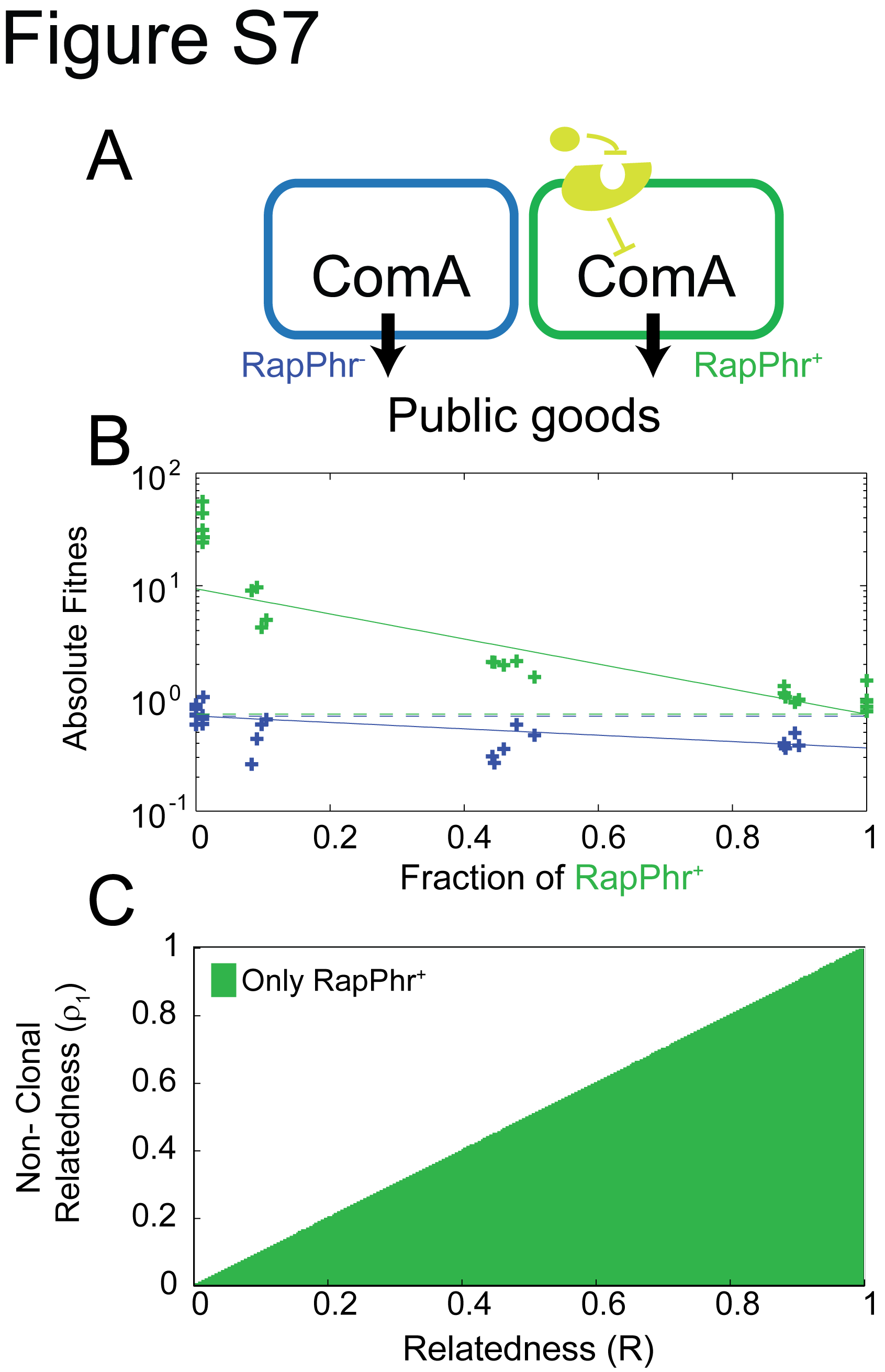

Supplement: Supplementary file 14 — Figure S7 [file 41396_2018_310_MOESM14_ESM.tif]
